# An analytical method to optimize residual stress in the ultrasonic rolling of titanium alloys Ti6Al4V

**DOI:** 10.1371/journal.pone.0351986

**Published:** 2026-06-26

**Authors:** Ho Thi My Nu, Phan Hoang Phung, Nguyen Vu Linh, Truyen Le

**Affiliations:** Ho Chi Minh city University of Industry and Trade, Tay Thanh Ward, Ho Chi Minh City, Tan Phu, Vietnam; University of Washington, UNITED STATES OF AMERICA

## Abstract

Ultrasonic rolling is an effective surface enhancement technique for titanium alloys because it introduces beneficial compressive residual stresses that improve fatigue resistance and service durability. In many aerospace and high-performance engineering applications, both the magnitude and penetration depth of the compressive residual stress field play critical roles in determining long-term structural reliability and resistance to crack initiation and propagation. However, the complex elastic–plastic deformation behavior induced by coupled static and ultrasonic loading makes accurate prediction and simultaneous optimization of these characteristics challenging. This study develops a unified analytical framework for simultaneously predicting and optimizing compressive residual stress magnitude and compressive layer depth during ultrasonic rolling of Ti6Al4V alloy. The proposed approach combines Hertzian contact mechanics, elastic–plastic deformation theory, and response surface methodology to systematically investigate the effects of static load, vibration amplitude, ultrasonic frequency, and rolling ball radius on residual stress evolution and hardened layer development. The analytical predictions were validated through comparison with experimentally reported residual stress profiles under different ultrasonic rolling conditions. The validation results demonstrate reasonable agreement between the analytical predictions and experimental observations in terms of residual stress distribution, peak compressive stress magnitude, and penetration depth. The parametric analysis indicates that static load and vibration amplitude exert dominant influences on residual stress evolution and compressive layer formation. The proposed framework provides a computationally efficient and physically interpretable methodology for analyzing and optimizing ultrasonic rolling parameters for Ti6Al4V alloy.

## 1. Introduction

Conventional techniques for enhancing surface strength, such as shot peening, laser shock peening, liquid jet peening, and low-plasticity burnishing, induce compressive residual stresses that improve the wear resistance, fatigue endurance, and ductility of titanium components. Nonetheless, conventional approaches have several drawbacks, including significant plastic deformation, superficial layer depth, limited processing efficiency, and imprecise processing trajectories [1]. Ultrasonic vibration-assisted rolling has recently emerged as an effective method for surface treatment that preserves advantageous compressive residual stresses and enhances surface integrity during roughing or semi-finishing [2].

Ultrasonic surface rolling technology (URT) has gained prominence in the automotive, aerospace, and energy sectors because of its ability to enhance performance and longevity of components [3,4]. In the automotive sector, URT minimizes wear on engines, gearboxes, and shafts operating at high load and speed [5]. This technology is utilized in the aerospace sector to prevent the failure and deterioration of turbine blades, landing gear, and structural components exposed to high pressure and cyclic stresses. Ultrasonic surface treatment benefits power generation systems, oil and gas turbine rotors, pressure vessels, and pipelines in the energy sector by mitigating stress corrosion cracking, thermal fatigue, and cyclic stress. URT is deemed highly appropriate for high-performance, safety-critical engineering applications because of these characteristics. It enhances safety and dependability while reducing maintenance expenses and downtime [6,7]. Residual stress is essential for preventing stress corrosion cracking, since it impedes crack initiation and bolsters material strength [8]. It directly affects the durability, robustness, and load-bearing ability of engineering components. To ensure long-term reliability, it is crucial to accurately measure and alleviate residual stress [9]. Multiple studies demonstrate that the ultrasonic rolling process (URP) substantially affects the integrity of the material’s surface, along with its resistance to fatigue, corrosion, and wear. This is because URP enhances the strength and environmental resilience of the treated material through the induction of controlled compressive residual stress and the refinement of the surface grain structure. Ultrasonic rolling has demonstrated a considerable improvement in surface homogeneity, augmented deformation, and an increase in the thickness of the residual compressive stress layer. The combination of static pressure and ultrasonic vibration causes substantial plastic deformation, promoting the refinement of underlying grains and the production of a thick nanocrystalline surface layer. This technique enhances wear and corrosion resistance while augmenting fatigue performance by stabilizing surface compressive stress. Ultrasonic rolling yields a more homogeneous and finer surface morphology than traditional mechanical rolling or shot peening. This makes it an efficient technique for producing high-performance engineering alloys [1,5,7,8,10].

In recent years, the Ti6Al4V titanium alloy has been extensively utilized in aerospace, biomedical, and high-performance mechanical applications owing to its superior strength-to-weight ratio, excellent corrosion resistance, and biocompatibility. Within these safety-critical sectors, mitigating surface defects and enhancing surface integrity are paramount, as they directly dictate the fatigue strength, long-term durability, and structural reliability of vital components subject to cyclic mechanical loading. However, Ti6Al4V has poor thermal conductivity and limited plasticity at ambient temperature, potentially resulting in machining inaccuracies, including micro-cracks and tensile residual stresses [11]. To address these constraints, rolling treatments—particularly surface rolling and ultrasonic surface rolling—have been devised to improve surface integrity and mechanical performance [12]. The combined effects of severe plastic deformation, strain hardening, and the introduction of residual compressive stress enhance the fatigue strength, wear resistance, and longevity of Ti6Al4V components by rolling [13]. The rolling process generates compressive contact force and tangential shear, resulting in significant plastic strain on the surface vicinity. This initiates dynamic recovery and recrystallization processes, resulting in ultrafine or nanocrystalline grains [14]. The increased density of dislocations enhances the material’s hardness and reduces its susceptibility to fracture. The compressive stress field generated beneath the surface prevents the occurrence of fractures during load cycling [15]. The enhanced grain structure further augments tribological performance and corrosion resistance by stabilizing surface oxides and reducing surface roughness. Ultrasonic-assisted rolling employs high-frequency vibrations to improve plastic flow, diminish frictional heating, and augment the penetration depth of the compressive stress layer relative to conventional rolling methods. Ultrasonic rolling treatment significantly improves the surface integrity, fatigue resistance, and long-term durability of Ti6Al4V components, rendering it an invaluable post-processing technique for aerospace and biomedical applications [16].

During ultrasonic rolling, the cyclic plastic deformation process is highly complex due to the combined effects of static contact loading and high-frequency ultrasonic vibration. The rolling ball undergoes nonlinear dynamic interaction with the material surface, resulting in complex stress redistribution and residual compressive stress formation within the near-surface region. Experimental studies have shown that process parameters such as static load, vibration amplitude, ultrasonic frequency, and rolling ball diameter strongly influence the resulting residual stress distribution and hardened layer thickness [16–18]. Depending on the selected parameter combinations, these factors may produce either beneficial or unfavorable residual stress states.

In many aerospace and high-performance engineering applications, both the magnitude of compressive residual stress and the penetration depth of the compressive stress layer play critical roles in determining fatigue resistance and long-term structural reliability. For example, Ti6Al4V is widely used in turbine blades, landing gear components, aircraft fasteners, biomedical implants, and high-speed rotating shafts, where components are frequently subjected to cyclic loading and subsurface crack propagation. In such applications, a high compressive residual stress confined only to a shallow surface region may not provide sufficient resistance against crack initiation and crack growth under long-term service conditions. Therefore, simultaneous control of both compressive residual stress magnitude and compressive layer depth is essential for effective ultrasonic rolling process optimization and long-term fatigue performance enhancement.

Despite extensive experimental and numerical investigations on ultrasonic rolling of titanium alloys, most existing studies rely primarily on empirical observations or computationally expensive finite-element simulations within limited parameter ranges. Furthermore, previous research has mainly focused on qualitative trend analysis rather than systematic analytical prediction and optimization of residual stress characteristics. The cyclic and nonlinear nature of ultrasonic-assisted contact deformation also makes it difficult to establish generalized analytical relationships between process parameters and residual stress evolution.

To address these limitations, the present study develops a unified analytical framework for ultrasonic rolling of Ti6Al4V alloy based on Hertzian contact mechanics and elastic–plastic deformation theory. Unlike conventional empirical approaches, the proposed framework enables simultaneous analytical prediction of both compressive residual stress magnitude and compressive layer depth under coupled static and ultrasonic loading conditions. In addition, a Design of Experiments (DoE) approach combined with response surface methodology (RSM) is employed to systematically evaluate the effects of process parameters and their interaction effects on residual stress evolution.

The proposed methodology provides a computationally efficient and physically interpretable framework for analyzing and optimizing ultrasonic rolling parameters for Ti6Al4V alloy. The analytical predictions are further validated through comparison with experimentally reported residual stress profiles available in the literature.

## 2. Analytical model

**Fig 1:**
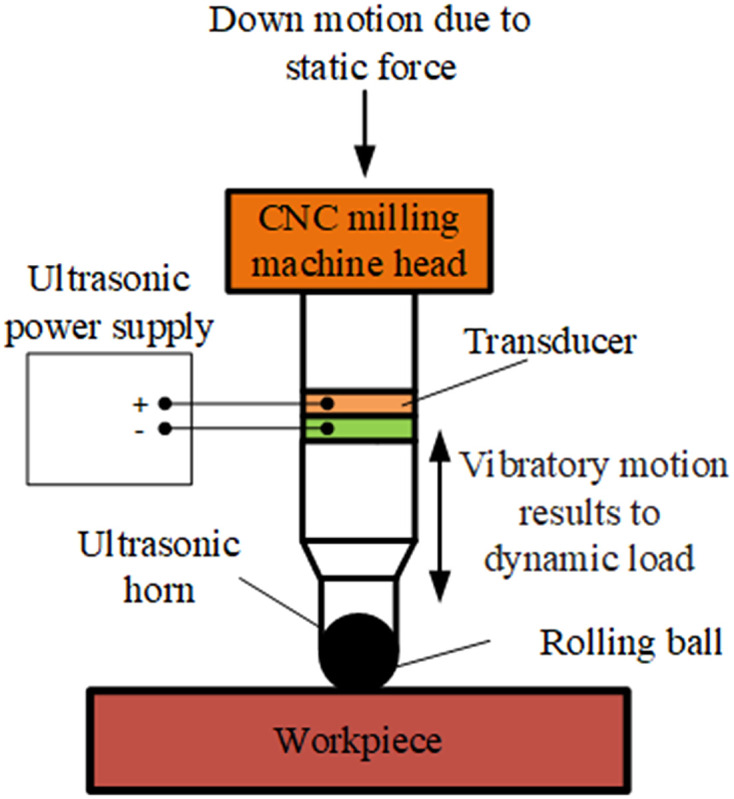
Principal diagram of ultrasonic rolling processing.

The ultrasonic surface rolling process is primarily affected by the synergistic effects of static pressure and ultrasonic impact force on surface enhancement as shown in Fig 1. The ultrasonic rolling system primarily consists of the ultrasonic power supply, transducer, horn, and rolling ball.

The continuous static pressure exerted by the rolling tool guarantees uniform and close contact between the ball and the workpiece surface. This pressure causes localized plastic deformation, resulting in the creation of compressive residual stress fields and a notable enhancement in surface integrity, hardness, and fatigue resistance [19]. The ultrasonic impact dynamic force, generated by the tool tip’s high-frequency vibrations, superimposes on the static load. The oscillatory effects induce cyclic high-strain-rate deformation in the top layer, promoting dynamic recovery and dynamic recrystallization processes. As a result, the near-surface microstructure experiences grain refinement, leading to improved hardness and wear resistance. The interplay of these two pressures generates a distinct dual effect: static pressure regulates macroscopic plastic flow and residual stress buildup, whereas ultrasonic impact predominantly induces microstructural alterations via energy-intensive dynamic deformation. This combination enables USRP to produce surfaces with enhanced mechanical qualities compared to those achieved by traditional mechanical rolling or shot peening [19]. This work examines the distinct impacts of the static loading component and the impact force produced by the tool’s ultrasonic vibration to provide a dependable deformation model for the ultrasonic surface rolling process.

### 2.1. Loading in ultrasonic rolling

**Fig 2:**
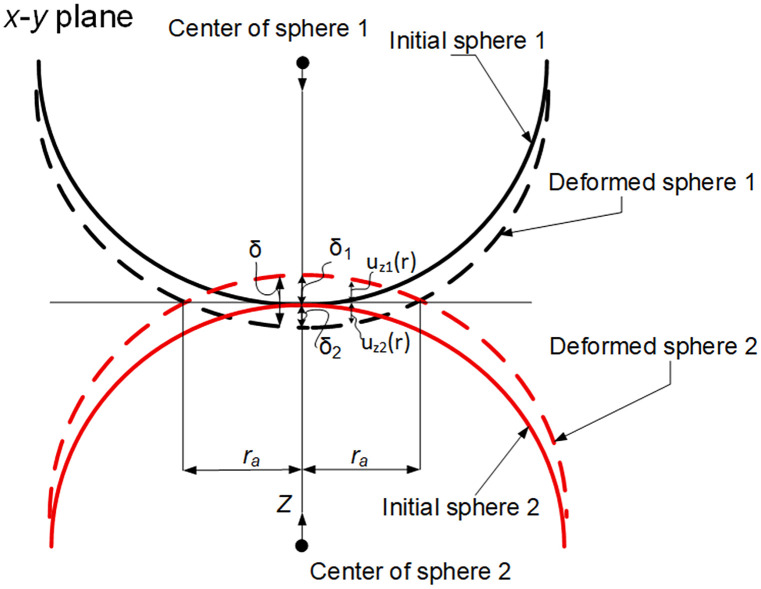
Geometry of contacting surfaces.


The impact zone undergoes plastic deformation, whereas the target is modeled as an elastic-plastic body. As illustrated in Fig 2, the boundary condition for normal displacements in the circular contact zone of two interfacing spheres is [20]:

$$\begin{array}{c} u_{z}(r)=u_{z\text{1}}(r)+u_{z\text{2}}(r)=\delta -\left(\frac{\text{1}}{\text{2}R}\right)r^{\text{2}} \end{array}$$ (1)

In the above scenario, $u_{z\text{1}}(r)$ and $u_{z\text{2}}(r)$ denote the normal displacement fields on the surfaces of the two spheres, respectively, *r* indicates the radial distance from the contact center, and *δ* represents the total approach between the centers of the two spheres, defined as *δ* = *δ*_1_ + *δ*_2_, where *δ*_1_ and *δ*_2_ are the displacements of the centers of the respective spheres. *R* signifies the relative radius of the interface, determined by the equation:


$$\begin{array}{c} \left(\frac{\text{1}}{R}\right)=\left(\frac{\text{1}}{R_{\text{1}}}\right)+\left(\frac{\text{1}}{R_{\text{2}}}\right) \end{array}$$ (2)

where *R*_1_ and *R*_2_ signify the initial radius of the two spheres, respectively.

The force *F* acting on the workpiece produces compressive pressure *p*(*r*). The pressure distribution *p*(*r*) proposed by Hertz for two frictionless elastic bodies in rolling contact may be expressed as. [20]:


$$\begin{array}{c} p(r)=p_{\text{0}}{\left[\text{1}-{\left(\frac{r}{r_{a}}\right)}^{\text{2}}\right]}^{\text{1}/\text{2}} \end{array}$$ (3)

The positive displacement produced by contact pressure *p*(*r*) can be calculated as:


$$\begin{array}{c} u_{z}(r)=\frac{\pi p_{\text{0}}}{\text{4}E_{eq}r_{a}}\left(\text{2}\overset{\text{2}}{\underset{a}{r}}-r^{\text{2}}\right),r<r_{a} \end{array}$$ (4)

where *p*_0_ denotes the maximum pressure at *r* = 0, *r*_*a*_ is the contact radius and *E*_*eq*_ represents the equivalent elasticity modulus of contact between two bodies and it can be computed as follows:


$$\begin{array}{c} \frac{\text{1}}{E_{eq}}=\frac{\text{1}-\overset{\text{2}}{\underset{\text{1}}{\vartheta }}}{E_{\text{1}}}+\frac{\text{1}-\overset{\text{2}}{\underset{\text{2}}{\vartheta }}}{E_{\text{2}}} \end{array}$$ (5)

where *E*_1_ and *E*_2_ denote the modulus of elasticity of the sheet and ball, respectively; and *ϑ*_1_ and *ϑ*_2_ represent the Poisson’s ratio of the sheet and ball, respectively.

The Hertz contact theory is employed to model the static and dynamic load components applied by the ultrasonic instrument on the workpiece.

#### 2.1.1. For static load.

By combining Eqs. (1) and (4) with the note that the depth $\delta_{es}$ is caused by the static pressure $p_{s}$ induced by static force, we obtain:


$$\begin{array}{c} \frac{\pi p_{s}}{\text{4}E_{eq}r_{s}}\left(\text{2}\overset{\text{2}}{\underset{s}{r}}-r^{\text{2}}\right)=\delta_{es}-\left(\frac{\text{1}}{\text{2}R}\right)r^{\text{2}} \end{array}$$ (6)

where *r*_*es*_ is the radius of the elastic deformation zone caused by static force.

It is noted that, when *r* = *r*_*es*_ then $u_{z}(r)=\text{0}$, we derive from Eq. (6) that:


$$\begin{array}{c} \delta_{es}=\frac{\pi {r_{es}p}_{s}}{\text{2}E_{eq}} \end{array}$$ (7)

$$\begin{array}{c} r_{es}=\frac{\pi p_{s}R}{\text{2}E_{eq}} \end{array}$$ (8)

Considering the circular geometry of the contact area and the radial variation in contact pressure, the static contact force may be determined by integrating the contact pressure over the contact area element., *i.e.,* $F_{s}=\int p_{s}(r)dA$ Simultaneously, $A=\pi r^{\text{2}}$, therefore:


$$\begin{array}{c} F_{s}=\int_{\text{0}}^{r_{s}}p_{s}(r)\text{2}\pi rdr=\frac{\text{2}}{\text{3}}\pi \overset{\text{2}}{\underset{es}{r}}p_{s} \end{array}$$ (9)

The highest contact pressure $p_{s}$and the highest elastic contact radius $r_{es}$ can be derived by putting Eq. (8) into Eq. (9). Thus, $p_{s}$ and *r*_*es*_ can be expressed as follows:


$$\begin{array}{c} p_{s}=\frac{\text{1}}{\pi }\sqrt[{\text{3}}]{\frac{\text{6}F_{s}\overset{\text{2}}{\underset{eq}{E}}}{R^{\text{2}}}} \end{array}$$ (10)

$$\begin{array}{c} r_{es}=\sqrt[{\text{3}}]{\frac{\text{3}F_{s}R}{\text{4}E_{eq}}} \end{array}$$ (11)

The static contact force is determined based on the displacement of the center in Eq. (7) and the center of contact in Eq. (11) as follows:


$$\begin{array}{c} F_{s}=\frac{\text{4}}{\text{3}}E_{eq}R^{\frac{\text{1}}{\text{2}}}{\delta_{es}}^{\frac{\text{3}}{\text{2}}} \end{array}$$ (12)

From Eqs. (7) and (8) we get:


$$\begin{array}{c} r_{es}=\sqrt{\delta_{es}R} \end{array}$$ (13)

#### 2.1.2. For dynamic load.

Analogous to static load, the dynamic load produced by the instruments ultrasonic vibration also induces elastic deformation in the workpiece. The vibration displacement may be calculated as follows:


$$\begin{array}{c} x=A_{\text{0}}\text{sin}(\text{2}\pi ft) \end{array}$$ (14)

where *A*_0_ denotes the amplitude of transverse ultrasound vibration, whereas *f* represents the frequency of ultrasonic vibration.

The velocity of the tool tip is derived as:


$$\begin{array}{c} v=\frac{dx}{dt}=\text{2}\pi fA_{\text{0}}cos(\text{2}\pi ft) \end{array}$$ (15)

So, the maximum speed of the tool head is:


$$\begin{array}{c} v_{max}=\text{2}\pi fA_{\text{0}} \end{array}$$ (16)

When the tool head impacts the material, this impact force will cause the material to sink a depth of *δ*_*ed*_. To determine the elastic contact radius and maximum contact pressure due to dynamic loading, one must consider the principles of work and energy:


$$\begin{array}{c} \frac{\text{1}}{\text{2}}mv_{max}=\int_{\text{0}}^{\delta_{ed}}F_{d}d\delta \end{array}$$ (17)

where *m* is the mass of the tool, i.e., $m=\left(\frac{\text{4}}{\text{3}}\right)\overset{\text{3}}{\underset{\text{1}}{R}}\pi \rho$ The dynamic force *F*_*d*_ **i**nduces an elastic penetration depth *δ*_*ed*_, and by utilizing the derived formula in Eq. (12) and using *R*_1_ = *R*, since $\left(R_{\text{2}}=\infty \right)$, Eq. (17) is reformulated as follows:


$$\begin{array}{c} \frac{\text{1}}{\text{2}}mv_{max}=\int_{\text{0}}^{\delta_{ed}}\left(\frac{\text{4}E_{eq}}{\text{3}}\right)R^{\frac{\text{1}}{\text{2}}}\delta^{\frac{\text{3}}{\text{2}}}d\delta \end{array}$$ (18)

Substitute *v*_*max*_ from Eq. (16) into Eq. (18) and solve the integral on the right side of Eq. (18), the following expression will be obtained:


$$\begin{array}{c} \delta_{ed}={\left(\frac{\text{5}\rho \pi^{\text{3}}{\overset{\text{3}}{\underset{\text{1}}{R}}f}^{\text{2}}\overset{\text{2}}{\underset{\text{0}}{A}}}{{R^{\frac{\text{1}}{\text{2}}}E}_{eq}}\right)}^{\frac{\text{2}}{\text{5}}} \end{array}$$ (19)

where ρ represents the density of the material utilized in the fabrication of the rolling ball.

According to Eq. (13), the elastic contact radius due to dynamic force can be expressed by the following relationship:


$$\begin{array}{c} r_{ed}=\sqrt{\delta_{ed}R}=\overset{\frac{\text{1}}{\text{2}}}{\underset{ed}{\delta }}.R^{\frac{\text{1}}{\text{2}}} \end{array}$$ (20)

Substitute Eq. (19) into Eq. (20) to get:


$$\begin{array}{c} r_{ed}={\left[\frac{\text{5}\rho \pi^{\text{3}}{\overset{\text{3}}{\underset{\text{1}}{R}}R^{\text{2}}f}^{\text{2}}\overset{\text{2}}{\underset{\text{0}}{A}}}{E_{eq}}\right]}^{\frac{\text{1}}{\text{5}}} \end{array}$$ (21)

The accumulated elastic contact radius in ultrasonic rolling, which includes both static and dynamic loads, is as follows:


$$\begin{array}{c} r_{e}=r_{es}+r_{ed} \end{array}$$ (22)

The compressive pressure caused by the dynamic load can be calculated as the formulas obtained in the static pressure stage. So, from Eq. (8) it follows that:


$$\begin{array}{c} p_{d}=\frac{\text{2}E_{eq}}{\pi R}r_{ed} \end{array}$$ (23)

where *p*_*d*_ is the maximum compressive pressure caused by the dynamic force.

Subsequently, replace Eq. (21) in Eq. (23) to obtain:


$$\begin{array}{c} p_{d}=\frac{\text{1}}{\pi }{\left[\frac{\text{1}\text{6}\text{0}\rho \pi^{\text{3}}{\overset{\text{3}}{\underset{\text{1}}{R}}f}^{\text{2}}\overset{\text{2}}{\underset{\text{0}}{A}}\overset{\text{4}}{\underset{eq}{E}}}{R^{\text{3}}}\right]}^{\frac{\text{1}}{\text{5}}} \end{array}$$ (24)

Thus, the total contact pressure on the workpiece surface is determined by summing the contact pressures from static and dynamic loads, i.e., *p*_0_ = *p*_*s*_ + *p*_*d*_.

### 2.2. Stress and strain during the elastic contact phase

In the elastic contact phase, the first interaction between the rolling ball and the workpiece transpires at a single point of contact. As the rolling force increases, point contact gradually evolves into surface contact. Due to the restricted dimensions of the contact surface in respect to the workpiece, the interacting entity may be regarded as a half-space body.

The contact surface in this phase is a circle with radius *r*_*e*_, which includes both static and impact forces as shown in Eq. (22). According to the Hertzian contact theory, the Hertzian contact stress generated by the roller on the workpiece surface penetrates inward into the workpiece. The interaction stress may be determined as [20]:


$$\begin{array}{c} \overset{e}{\underset{x}{\sigma }}(z)=-\overset{e}{\underset{y}{\sigma }}(z)=-p_{\text{0}}\left(\left(\text{1}+\vartheta_{\text{1}}\right)\left(\text{1}-\frac{z}{r_{e}}{tan}^{-\text{1}}\left(\frac{r_{e}}{z}\right)\right)+\frac{\text{1}}{\text{2}}{\left(\text{1}+\frac{z^{\text{2}}}{\overset{\text{2}}{\underset{e}{r}}}\right)}^{-\text{1}}\right) \end{array}$$ (25)

$$\begin{array}{c} \overset{e}{\underset{z}{\sigma }}(z)=-p_{\text{0}}{\left(\text{1}+\frac{z^{\text{2}}}{\overset{\text{2}}{\underset{e}{r}}}\right)}^{-\text{1}} \end{array}$$ (26)

where *σ* signifies the elastic stress components and *z* indicates the distance from the surface in the thickness direction.

The analogous von-Mises elastic stress and strain may be obtained as previously described.


$$\begin{array}{c} \overset{e}{\underset{i}{\sigma }}=\frac{\sqrt{\left({\left(\overset{e}{\underset{x}{\sigma }}-\overset{e}{\underset{y}{\sigma }}\right)}^{\text{2}}+{\left(\overset{e}{\underset{y}{\sigma }}-\overset{e}{\underset{z}{\sigma }}\right)}^{\text{2}}+{\left(\overset{e}{\underset{z}{\sigma }}-\overset{e}{\underset{x}{\sigma }}\right)}^{\text{2}}\right)}}{\sqrt{\text{2}}} \end{array}$$ (27)

The relevant strain $\overset{e}{\underset{i}{\varepsilon }}$ may be directly obtained from Hooke’s law as follows:


$$\begin{array}{c} \overset{e}{\underset{i}{\varepsilon }}=\frac{\overset{e}{\underset{i}{\sigma }}}{E_{\text{1}}} \end{array}$$ (28)

### 2.3. Stress and strain during the plastic contact phase

The establishment of accurate stress and strain relationships in elastic-plastic distortion interaction requires a sophisticated theoretical model. Li et al. [21] established a linear correlation between elasticity and elastic-plastic strain. His theory posits that within the elasticity-plastic interaction framework, the elasticity-plastic strain is determined by a linear relationship that encompasses both the purely elastic strain and the wholly plastic strain. When the stress level is below the yield stress, the plastic strain is zero, and the overall strain equates to the elastic strain. Beyond the yield point, the elastic-plastic phase commences; under these conditions, the elastic-plastic behavior can be approximated by the elastic strain as follows:


$$\begin{array}{c} \overset{p}{\underset{i}{\varepsilon }}=\left\{ \begin{array}{cc} \overset{e}{\underset{i}{\varepsilon }} & \overset{e}{\underset{i}{\varepsilon }}<e_{y}\\ \varepsilon_{y}+\psi \left(\overset{e}{\underset{i}{\varepsilon }}-\varepsilon_{y}\right) & \overset{e}{\underset{i}{\varepsilon }}>e_{y} \end{array}\right. \end{array}$$ (29)

where $\overset{p}{\underset{i}{\varepsilon }}$ signifies the cumulative elastic-plastic strain, obtained from the aggregation of elastic strain and perfectly plastic strain. In this equation, *ε*_*y*_ denotes the strain at yield stress, whereas *ψ* signifies the ratio of the maximum plastic contact radius *r*_*p*_ to the maximum elastic contact radius *r*_*e*_.


$$\begin{array}{c} \psi =\frac{r_{p}}{r_{e}} \end{array}$$ (30)

The elastic contact radius of *r*_*e*_ is determined by Eq. (22). Thus, in order to determine the coefficient ψ, the maximum plastic contact radius of the indentation $r_{p}$ needs to be evaluated. For this purpose, the kinematics of the ultrasonic surface rolling process are decomposed into two components: the static load *F*_*s*_ and the dynamic load *F*_*d*_, the latter corresponding to the ultrasonic vibration impact. Both components contribute to plastic deformation within a circular contact region characterized by the radius $r_{p}$.

#### 2.3.1. For static load.

Rolling produces a circular indentation between the rolling ball and the workpiece. Consequently, the maximum plastic radius *r*_*sp*_ in rolled plastic contact caused by static force is:


$$\begin{array}{c} F_{s}=p_{avg(s)}.A_{sp}=p_{avg(s)}\pi \overset{\text{2}}{\underset{sp}{r}} \end{array}$$ (31)

In which the average pressure of the resistance force in the contact region due to of static force is $p_{avg(s)},$ which is roughly 3*σ*_*s*_ [22].

**Fig 3:**
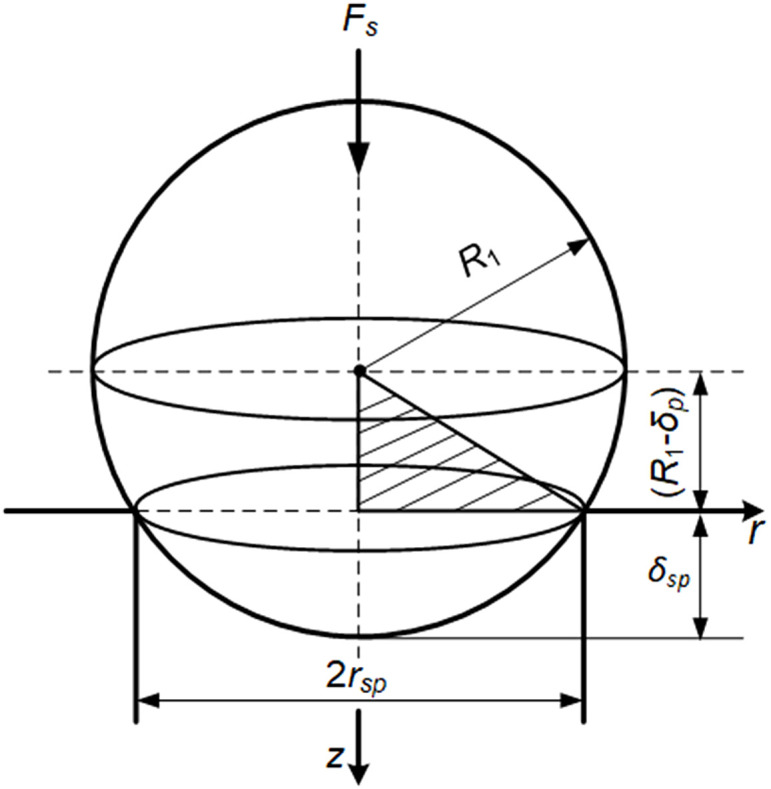
Plastic deformation of a rigid shot contacting a completely plastic target.


Fig 3 illustrates the relationship between deep penetration depth *δ*_*sp*_ and the maximum plastic contact radius *r*_*sp*_ inside the contact area induced by the static force as [15]:

$$\begin{array}{c} \overset{\text{2}}{\underset{\text{1}}{R}}={\left(R_{\text{1}}-\delta_{sp}\right)}^{\text{2}}+\overset{\text{2}}{\underset{sp}{r}} \end{array}$$ (32)

If *R*_1_ ≫ *δ*_*sp*_, Eq. (32) disregards $\overset{\text{2}}{\underset{sp}{\delta }}$. We get Eq. (33) as:


$$\begin{array}{c} r_{sp}=\sqrt{\text{2}R_{\text{1}}\delta_{sp}} \end{array}$$ (33)

By substituting Eq. (33) into Eq. (31), the penetration depth *δ*_*sp*_ is derived as follows:


$$\begin{array}{c} \delta_{sp}=\frac{F_{s}}{\text{6}\sigma_{s}\pi R_{\text{1}}} \end{array}$$ (34)

Using Eq. (20) and Eq. (34), the greatest plastic contact radius *r*_*sp*_ caused by static force may be represented as:


$$\begin{array}{c} r_{sp}=\sqrt{\frac{F_{s}}{\text{3}\pi \sigma_{s}}} \end{array}$$ (35)

#### 2.3.2. For dynamic load.

The geometric characteristics of the plastic indentation induced by a rigid sphere impacting an elastic-plastic target can be determined using the following formula:


$$\begin{array}{c} \left(\frac{\text{4}\pi }{\text{3}}\rho \overset{\text{3}}{\underset{\text{1}}{R}}\right)\frac{dv}{dt}=p_{avg(d)}.A_{dp}=p_{avg(d)}\pi {\left(r_{dp}\right)}^{\text{2}} \end{array}$$ (36)

Utilizing *δ*_*dp*_ as the comprehensive distance between the centers of the two entities, thus $v=\frac{d\delta_{dp}}{dt}$ and $\left(\frac{dv}{dt}\right)=\left(\frac{dv}{d\delta_{dp}}\right).\left(\frac{d\delta_{dp}}{dt}\right)=v.(dv/dt)$.

Thus:


$$\begin{array}{c} \frac{\text{4}\pi }{\text{3}}\rho \overset{\text{3}}{\underset{\text{1}}{R}}vdv=p_{avg(d)}\pi \left(\overset{\text{2}}{\underset{dp}{r}}\right)d\delta_{dp} \end{array}$$ (37)

According to Eq. (33), the plastic contact radius caused by the dynamic force is calculated as:


$$\begin{array}{c} r_{dp}=\sqrt{\text{2}R_{\text{1}}\delta_{dp}} \end{array}$$ (38)

Substitute Eq. (38) into Eq. (37), then compute the integral to obtain:


$$\begin{array}{c} \left(\frac{\text{4}}{\text{6}}\right)\pi \overset{\text{2}}{\underset{\text{1}}{R}}\rho v^{\text{2}}=p_{avg(d)}\overset{\text{2}}{\underset{dp}{\delta }} \end{array}$$ (39)

The compressive pressure caused by the impact dynamic force remains constant and equal to 3*σ*, thus:


$$\begin{array}{c} \delta_{dp}=\sqrt{\frac{\text{2}\rho \overset{\text{2}}{\underset{\text{1}}{R}}v^{\text{2}}}{\text{9}\sigma_{s}}} \end{array}$$ (40)

The maximum velocity is $v_{max}=\text{2}\pi A_{\text{0}}f$, so the depth caused by the impact dynamic force can be calculated as:


$$\begin{array}{c} \delta_{dp}=\sqrt{\frac{\text{2}\rho \overset{\text{2}}{\underset{\text{1}}{R}}{\left(\text{2}\pi A_{\text{0}}f\right)}^{\text{2}}}{\text{9}\sigma_{s}}}=\pi {R_{\text{1}}A}_{\text{0}}f\sqrt{\frac{\text{8}\rho }{\text{9}\sigma_{s}}} \end{array}$$ (41)

Using Eq. (38) we get:


$$\begin{array}{c} r_{dp}=R_{\text{1}}{\left[\frac{\text{3}\text{2}\rho \pi^{\text{2}}\overset{\text{2}}{\underset{\text{0}}{A}}f^{\text{2}}}{\text{9}\sigma_{s}}\right]}^{\frac{\text{1}}{\text{4}}} \end{array}$$ (42)

The plastic deformation zone, defined by the contact radius $r_{p}$, is assumed to result from the superposition of the plastic regions produced by the static load and the ultrasonic impact load as:


$$\begin{array}{c} r_{p}=r_{sp}+r_{dp} \end{array}$$ (43)

Thus, after determining the *r*_*p*_, the coefficients of *ψ* in Eq. (30) may be calculated, and the elastic-plastic strain value is thereafter determined.

Furthermore, after the strains are established, the stresses may be calculated using the elastic–plastic stress–strain curves as follows [21]:


$$\begin{array}{c} \overset{p}{\underset{i}{\sigma }}=\left\{ \begin{array}{cc} \overset{e}{\underset{i}{\sigma }} & \overset{p}{\underset{i}{\varepsilon }}<\varepsilon_{s}\\ \sigma_{s}+H\left(\overset{p}{\underset{i}{\varepsilon }}-\varepsilon_{s}\right) & \varepsilon_{s}<\overset{p}{\underset{i}{\varepsilon }}<\varepsilon_{b}\\ \sigma_{b} & \overset{p}{\underset{i}{\varepsilon }}\geq \varepsilon_{b} \end{array}\right. \end{array}$$ (44)

where *H* = (*σ*_*b*_-*σ*_*s*_)/(*ε*_*b*_-*ε*_*s*_) represents a linear stress-hardening parameter, *σ*_*b*_ denotes the maximum tensile stress of the material in question, *ε*_*b*_ symbolizes the strain corresponding to *σ*_*b*_, and *ε*_*s*_ indicates the elastic strain related to yield stress, calculated as follows:


$$\begin{array}{c} \varepsilon_{s}=\frac{\sigma_{s}}{E_{\text{1}}} \end{array}$$ (45)

The hydrostatic stress operates independently of plastic deformation, according to the principles of elastic-plastic mechanics. It just modifies the object’s volume without altering its shape and does not affect the material’s yield strength. Thus, deviatoric stress is essential in the plastic deformation of the material. To get deviatoric stress, one must first calculate the deviatoric strain. The association between $\overset{e}{\underset{ij}{e}}$ and $\overset{e}{\underset{ij}{\varepsilon }}$ is anticipated to stay valid throughout the elastic-plastic region. Consequently, we conclude:


$$\begin{array}{c} \left\{ \begin{array}{c} @l\overset{p}{\underset{x}{e}}=\overset{p}{\underset{z}{e}}=\frac{\text{1}}{\text{3}}\left(\text{1}+\vartheta_{\text{1}}\right)\overset{p}{\underset{i}{e}}\\ \overset{p}{\underset{y}{e}}=-\frac{\text{2}}{\text{3}}\left(\text{1}+\vartheta_{\text{1}}\right)\overset{p}{\underset{i}{e}} \end{array}\right. \end{array}$$ (46)

Iliushin’s theory posits that the deviatoric plastic stress tensor is derived as follows:


$$\begin{array}{c} \overset{p}{\underset{ij}{S}}=\frac{\text{1}}{\left(\text{1}+\vartheta_{\text{1}}\right)}\frac{\overset{p}{\underset{i}{\sigma }}}{\overset{p}{\underset{i}{\varepsilon }}}\overset{p}{\underset{ij}{e}} \end{array}$$ (47)

Eqs. (46) and (47) provide the deviatoric plastic stress tensor:


$$\begin{array}{c} \overset{p}{\underset{x}{S}}=\overset{p}{\underset{z}{S}}=\frac{\text{1}}{\left(\text{1}+\vartheta_{\text{1}}\right)}\frac{\overset{p}{\underset{i}{\sigma }}}{\overset{p}{\underset{i}{\varepsilon }}}\overset{p}{\underset{x}{e}}=\frac{\text{1}}{\text{3}}\overset{p}{\underset{i}{\sigma }} \end{array}$$ (48)

$$\begin{array}{c} \overset{p}{\underset{y}{S}}=-\frac{\text{2}}{\text{3}}\overset{p}{\underset{i}{\sigma }}=-\text{2}\overset{p}{\underset{x}{S}} \end{array}$$ (49)

The subsequent section derives the induced stress on the target material upon unloading from the elastic–plastic stress and strain representations.

### 2.4. Residual stress post-unloading

It is essential to differentiate between transresidual stresses in the target material after the sequential loading and unloading of a single shot and the induced stresses resulting from complete shot peening coverage. The estimation of residual stress using ultrasonic rolling is predicated on the following assumptions. Isotropically hardened workpieces disregard the Bauschinger effect. Ultrasonic rolling induces plastic deformation instead of considerable distortion. Rebound remains elastic until reverse yielding transpires. As hydrostatic stress does not influence plastic deformation, only deviatoric stress may induce plastic deformation and residual stress inside the material. Determining residual stress in single-pass ultrasonic rolling [23]:


$$\begin{array}{c} \overset{r}{\underset{ij}{\sigma }}=\left\{ \begin{array}{c} @l\text{0}\;\overset{e}{\underset{i}{\sigma }}<\sigma_{s}\\ \overset{p}{\underset{i}{S}}-\overset{e}{\underset{i}{S}}\;\sigma_{s}\leq \overset{e}{\underset{i}{\sigma }}\leq \text{2}\overset{p}{\underset{i}{\sigma }} \end{array}\right. \end{array}$$ (50)

These interactions may be delineated along three principal axes: *x*, *y*, and *z* as follows:


$$\begin{array}{c} \overset{r}{\underset{x}{\sigma }}=\overset{r}{\underset{y}{\sigma }}=\frac{\text{1}}{\text{3}}\left(\overset{p}{\underset{i}{\sigma }}-\overset{e}{\underset{i}{\sigma }}\right),\sigma_{s}\leq \overset{e}{\underset{i}{\sigma }}\leq \text{2}\overset{p}{\underset{i}{\sigma }} \end{array}$$

$$\begin{array}{c} \overset{r}{\underset{z}{\sigma }}=-\text{2}\overset{r}{\underset{z}{\sigma }} \end{array}$$ (51)

When $\overset{e}{\underset{i}{\sigma }}$ exceeds $\text{2}\overset{p}{\underset{i}{\sigma }}$, the target materials will experience reverse yielding and hardening. Initially, a stress of $\text{2}\overset{p}{\underset{i}{\sigma }}$ is elastically discharged, subsequently leading to the onset of reverse yielding. However, some pressures may remain unmitigated, specifically:


$$\begin{array}{c} \Delta \overset{e}{\underset{i}{\sigma }}=\overset{e}{\underset{i}{\sigma }}-\text{2}\overset{p}{\underset{i}{\sigma }} \end{array}$$ (52)

The elastic strain associated with $\overset{e}{\underset{i}{\sigma }}$are:


$$\begin{array}{c} \Delta \overset{e}{\underset{i}{\varepsilon }}=\frac{\Delta \overset{e}{\underset{i}{\sigma }}}{E_{\text{1}}} \end{array}$$ (53)

Subsequently, analogous to the plastic–elasticity contact, the plastic–elasticity strain is expected to be:


$$\begin{array}{c} \Delta \overset{p}{\underset{i}{\varepsilon }}=\psi \Delta \overset{e}{\underset{i}{\varepsilon }} \end{array}$$ (54)

The related stress $\Delta \overset{p}{\underset{i}{\sigma }}$ may be obtained by examining the multilinear stress–strain curve of the material in question as follows:


$$\begin{array}{c} \Delta \overset{p}{\underset{i}{\sigma }}=H\Delta \overset{p}{\underset{i}{\varepsilon }} \end{array}$$ (55)

Equation (55) asserts that reversing yields negligible stress. In cases of reverse yielding under significant loads (e.g., from high-velocity impacts), $\Delta \overset{p}{\underset{i}{\sigma }}$ must be obtained from the elastic–plastic stress–strain curve, considering the effect of *σ*_*b*_ during reversed yielding. Residual stress may be obtained when $\overset{e}{\underset{i}{\sigma }}>\text{2}\overset{p}{\underset{i}{\sigma }}$ as follows


$$\begin{array}{c} \overset{r}{\underset{x}{\sigma }}=\overset{r}{\underset{y}{\sigma }}=\frac{\text{1}}{\text{3}}\left(\overset{p}{\underset{i}{\sigma }}-\overset{e}{\underset{i}{\sigma }}\right) \end{array}$$ (56)

$$\begin{array}{c} \overset{r}{\underset{z}{\sigma }}=-\text{2}\overset{r}{\underset{x}{\sigma }} \end{array}$$ (57)

Upon achieving full shot peening coverage (100% penetration), the deformation field is assumed to be steady and continuous. The target component is presumed to sustain a flat surface, indicating that *ε*_*x*_ and *ε*_*y*_ are null, whereas the positive stress and strain components will be distinct from *x* and *y*. Therefore, with total coverage:


$$\begin{array}{c} \sigma_{x}=\sigma_{y}=f(z) \end{array}$$ (58)

$$\begin{array}{c} \sigma_{z}=\text{0} \end{array}$$ (59)

$$\begin{array}{c} \varepsilon_{x}=\varepsilon_{y}=\text{0} \end{array}$$ (60)

$$\begin{array}{c} \varepsilon_{z}=f_{\text{1}}(z) \end{array}$$ (61)

To satisfy the specified equilibrium conditions, σi must be substantially mitigated. According to Hooke’s law, the relaxation stresses $\overset{rel}{\underset{x}{\sigma }}$ and $\overset{rel}{\underset{z}{\sigma }}$ may be expressed as:


$$\begin{array}{c} \overset{rel}{\underset{x}{\sigma }}=\overset{rel}{\underset{z}{\sigma }}=\frac{\text{1}}{\text{1}-\vartheta_{\text{1}}}\overset{r}{\underset{y}{\sigma }} \end{array}$$ (62)

The induced stress resulting from complete peening coverage (*σ*^*ind*^) may be calculated as:


$$\begin{array}{c} \overset{ind}{\underset{x}{\sigma }}=\overset{ind}{\underset{y}{\sigma }}=\overset{r}{\underset{x}{\sigma }}-\overset{rel}{\underset{x}{\sigma }}=\overset{r}{\underset{x}{\sigma }}-\frac{\vartheta_{\text{1}}}{\text{1}-\vartheta_{\text{1}}}\overset{r}{\underset{z}{\sigma }}=\frac{\text{1}+\vartheta_{\text{1}}}{\text{1}-\vartheta_{\text{1}}}\overset{r}{\underset{x}{\sigma }} \end{array}$$ (63)

$$\begin{array}{c} \overset{ind}{\underset{z}{\sigma }}=\text{0} \end{array}$$ (64)

The induced stresses are regarded as a dependable approximation of the produced stresses at saturation (in terms of Almen intensity) and thus be used to calculate theoretical Almen intensity.

## 3. Mathematical computation

### 3.1. Material and processing parameters

The dimensions of the Ti6Al4V specimen in this study were established according to the experimental setup outlined by Bozdana et al. [24]. The specimen is a cuboid with dimensions of 5 mm x 5 mm x 6 mm. The parameters employed in the ultrasonic surface rolling process for prediction of the residual stress distribution in the proposed analytical model were as follows: (a) an ultrasonic frequency of 20 kHz, a static load of 30 N, a rolling ball diameter of 6 mm, and a vibration amplitude of 8.72 μm; and (b) an ultrasonic frequency of 20 kHz, a static load of 380 N, a rolling ball diameter of 6 mm, and a vibration amplitude of 10.9 μm. These parameter values correspond to the experimental conditions reported by Bozdana et al. in their ultrasonic deep cold rolling study. The initial surface residual stress produced by the turning operation is −7.24 MPa.

Table 1 delineates the chemical composition of Ti6Al4V, whereas Table 2 specifies the mechanical properties of the workpiece and roller materials.

**Table 1:** Chemical composition of Ti6Al4V (wt.%).

Fe	C	N	H	O	Al	V	Ti
0.1	0.012	0.009	0.004	0.09	6.08	4.1	Bal.

**Table 2:** Mechanical properties of materials [24].

Materials	Density *ρ* (kg/m^3^)	Elastic modulus *E* (Gpa)	Poisson ratio *v*	Yield strength *σ*_*s*_ (Mpa)	Ultimate tensile stress *σ*_*b*_ (Mpa)	Strain *ε*_*s*_	Strain *ε*_*b*_
Ti6Al4v	4430	110	0.33	880	1078	0.8%	11%
YG8	15800	700	0.24				

The research approach is divided into three main steps. The first step is to check the proposed residual stress model against experimental data for TI6Al4V. The second stage focuses on looking at how important process parameters affect the distribution of residual stress in a parametric way. This stage entails executing a series of simulations based on the analytical model, employing a single-factor experimental design in which one process parameter is modified within a defined range, while all other parameters are held constant. This enables an individual evaluation of the impact of each parameter on the evolution and magnitude of the residual stress field. The third stage is to find the best settings for the rolling process to produce the best residual stress state. It also gives practical advice on how to improve the process and performance.

### 3.2 Results and discussion

#### 3.2.1. Validation of the model.

To validate the predictive capability of the proposed analytical framework for residual stress evolution during ultrasonic surface rolling, the analytical results were compared with the experimental residual stress distributions reported by Bozdana [24] for Ti6Al4V alloy subjected to ultrasonic deep cold rolling (UDCR). Two validation conditions corresponding to static loads of 30 N and 380 N were considered to evaluate the model performance under both low-force and high-force loading conditions.

**Fig 4:**
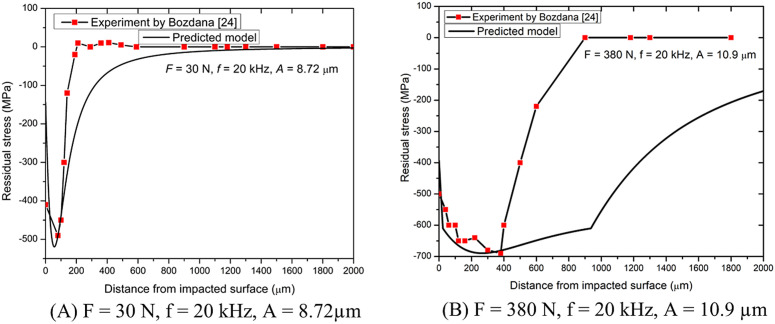
Comparison results between predictive model and experiment.

Fig 4 compares the analytically predicted residual stress distributions with the experimentally measured residual stress profiles reported by Bozdana [24]. The validation was conducted under two experimental conditions: (a) an ultrasonic frequency of 20 kHz, a static load of 30 N, a rolling ball diameter of 6 mm, and a vibration amplitude of 8.72 μm (Fig 4A); and (b) an ultrasonic frequency of 20 kHz, a static load of 380 N, a rolling ball diameter of 6 mm, and a vibration amplitude of 10.9 μm (Fig 4B).

For both loading conditions, the proposed analytical framework reasonably reproduces the experimentally observed residual stress evolution beneath the treated surface. The predicted results successfully capture the formation of the near-surface compressive residual stress (CRS) region as well as the general depth-dependent stress evolution trend. In both the analytical predictions and the experimental observations, the compressive residual stress gradually decreases with increasing depth and eventually approaches a nearly neutral stress state within the bulk material.

To further evaluate the predictive capability of the proposed analytical framework, additional quantitative error metrics were introduced based on comparisons between the analytical predictions and the experimentally observed residual stress profiles. The experimental residual stress data were extracted from the published Figs using the WebPlotDigitizer software.

Since the proposed analytical framework was primarily developed to predict the dominant characteristics of near-surface compressive residual stress evolution and compressive residual stress layer development, the quantitative error analysis was performed within the near-peak compressive residual stress region rather than at the immediate surface. The surface point at 0 μm was excluded from the quantitative error calculation because the largest local discrepancy occurred at the immediate surface region, where residual stress measurements are highly sensitive to experimental uncertainty, surface preparation conditions, data extraction accuracy, and local microstructural effects that are not fully represented in the simplified analytical formulation. Accordingly, the quantitative error analysis was conducted within the ranges of 80–100 μm for the 30 N condition and 100–400 μm for the 380 N condition, corresponding to the near-peak compressive residual stress region.

The root mean square error (RMSE) and mean absolute error (MAE) were calculated as follows:

RMSE


$$\begin{array}{c} RMSE=\sqrt{\frac{\text{1}}{n}\sum_{i=\text{1}}^{n}{\left(\sigma_{pred,i}-\sigma_{exp,i}\right)}^{\text{2}}} \end{array}$$ (65)

MAE


$$\begin{array}{c} MAE=\frac{\text{1}}{n}\sum_{i=\text{1}}^{n}\left|\sigma_{pred,i}-\sigma_{exp,i}\right| \end{array}$$ (66)

where $\sigma_{pred,i}$ and $\sigma_{exp,i}$ are the predicted and experimental residual stress values at the *i*^*th*^ depth location, respectively, and *n* is the number of comparison points.

Table 3 summarizes the quantitative comparison results between the proposed analytical model and the experimentally observed residual stress profiles reported by Bozdana [24].

**Table 3:** Quantitative comparison between the proposed analytical model and the experimental residual stress profiles reported by Bozdana.

Validation case	Depth range used for error analysis (µm)	Peak compressive residual stress (Exp.) [MPa]	Peak compressive residual stress (Pred.) [MPa]	Peak stress error (%)	Peak depth (Exp.) [µm]	Peak depth (Pred.) [µm]	Depth error (%)	RMSE (MPa)	MAE (MPa)
UDCR 30 N	80-100	−490	−490	0.00	80	80	0.00	7.78	5.50
UDCR 380 N	100-400	−690	−689	0.14	380	300	21.05	47.51	38.00

For the 380 N condition, the analytical prediction gives a peak compressive residual stress of approximately −689 MPa at a depth of about 300 μm, compared with the experimentally observed value of approximately −690 MPa at a depth close to 380 μm. The corresponding peak stress and peak depth errors are approximately 0.14% and 21.05%, respectively. The RMSE and MAE values within the investigated near-peak compressive residual stress region are 47.51 MPa and 38.00 MPa, respectively.

For the 30 N condition, the predicted peak compressive residual stress and corresponding peak depth show good agreement with the experimentally observed values, with both the peak stress error and peak depth error equal to 0.00%. The corresponding RMSE and MAE values within the investigated near-peak region are 7.78 MPa and 5.50 MPa, respectively.

The obtained quantitative comparison results indicate that the proposed analytical framework can reasonably reproduce the dominant characteristics of near-surface residual stress evolution and compressive layer development during ultrasonic rolling of Ti6Al4V alloy within the investigated parameter range.

The remaining discrepancies between the analytical predictions and the experimental profiles may be associated with the simplifying assumptions adopted in the analytical formulation, including isotropic hardening behavior, simplified representation of the coupled static–dynamic loading condition, omission of strain-rate-dependent constitutive effects, and uncertainties associated with extraction of experimental data from published Figs.

The proposed analytical framework adopts several simplifying assumptions to maintain computational efficiency and analytical tractability, including isotropic hardening behavior, neglect of the Bauschinger effect, simplified representation of the coupled static–dynamic loading condition, and omission of strain-rate-dependent constitutive effects and thermal influences. Although these simplifications may affect prediction accuracy under highly nonlinear cyclic deformation conditions associated with ultrasonic rolling, the proposed model still demonstrates reasonable agreement with experimentally observed residual stress profiles and reasonably captures the dominant trends governing residual stress evolution during ultrasonic rolling. Although some discrepancies remain between the analytical predictions and the experimentally observed residual stress profiles, particularly near the immediate surface region, the obtained quantitative error metrics demonstrate that the proposed analytical framework can reasonably capture the dominant characteristics of compressive residual stress evolution and compressive layer development during ultrasonic rolling of Ti6Al4V alloy. The obtained RMSE and MAE values within the near-peak compressive residual stress region, together with the good agreement in peak compressive residual stress magnitude and penetration depth, indicate that the proposed analytical model provides reasonable predictive capability for parametric analysis, process optimization, and preliminary engineering design applications. Considering the computational efficiency and physical interpretability of the analytical framework compared with computationally intensive finite-element simulations, the obtained prediction accuracy is considered acceptable within the scope of the present study.

#### 3.2.2. Design of experiments and response surface method.

Two main reaction characteristics that affect the ultrasonic surface rolling process are the surface residual compressive stress and the depth of the maximum hardened layer. Both responses contribute significantly to improving fatigue strength, delaying crack initiation, and enhancing component durability. A higher and more stable compressive stress makes it harder for fractures to spread, and a deeper hardened layer increases the area that can bear weight, which improves fatigue performance under tough or cyclic conditions. Focusing on only one of these responses is insufficient for achieving optimal surface performance. For example, a surface layer with high compressive stress but limited penetration depth may provide only short-term strengthening benefits, whereas a deep layer associated with insufficient compressive stress may provide limited fatigue improvement. Therefore, simultaneous optimization of compressive residual stress and hardened layer depth is essential for maintaining surface integrity and long-term reliability while achieving an effective balance between mechanical performance, fatigue resistance, and process feasibility. Preliminary investigations indicated that the residual stress profile and hardened depth are mostly affected by the static force *F*, vibration amplitude *A*, ball diameter *D*, and ultrasonic frequency *f*. So, these four traits were chosen to be independent factors in this study. The parameter domains were specified as *F* = 180–380 N, *A* = 6–16 µm, *D* = 4–6 mm, and *f* = 15–25 kHz.

A Box–Behnken Design (BBD) was employed to systematically examine the influence of these factors and to construct a second-order Response Surface Model (RSM). BBD guarantees statistical robustness with a limited number of tests, avoiding extreme factor combinations that might damage equipment or specimens. A total of 27 randomized trials were conducted, incorporating three coded levels for each variable: high (+1), medium (0), and low (–1). This experimental design enables the identification of main, quadratic, and interaction effects among the variables.

The resulting regression model for each response ($\hat{Y}$) is expressed as:


$$\begin{array}{c} \hat{Y}=\beta_{\text{0}}+\sum_{i=\text{1}}^{\text{4}}\beta_{i}X_{i}+\sum_{i<j}\beta_{ij}X_{i}X_{j}+\sum_{i=\text{4}}^{\text{4}}\beta_{ii}\overset{\text{2}}{\underset{i}{X}} \end{array}$$ (67)

where $\hat{Y}$ represents the response variable (either $\overset{R}{\underset{x}{\sigma }}$ or *z*_0_), $X_{i}$are the coded factors corresponding to *F*, *A*, *D*, and *f*, and β terms denote the intercept, linear, interaction, and quadratic coefficients.

This study will employ data from the analytical model instead of experimental results. The analytical model has been validated, establishing the credibility of the conclusions derived from it. Model adequacy and statistical significance were evaluated by Analysis of Variance (ANOVA), employing R² indices to validate regression and estimate goodness of fit. The coefficient of determination, R², may be articulated as:


$$\begin{array}{c} R^{\text{2}}=\text{1}-\frac{SSE}{SST} \end{array}$$ (68)

$$\begin{array}{c} SSE=\sum_{i=\text{1}}^{n}{\left(Y_{i}-{\hat{Y}}_{i}\right)}^{\text{2}};SST=\sum_{i=\text{1}}^{n}{\left(Y_{i}-\overset{―}{Y}\right)}^{\text{2}} \end{array}$$ (69)

Equation (67) demonstrates that many polynomial forms, such as linear, quadratic, and cubic functions, may be utilized to develop approximation models. This research utilized a comprehensive quadratic model for the response surface functions, attaining R² values exceeding 0.99 while avoiding overfitting the training dataset with higher-order polynomials.

Table 4 delineates the ranges of variables and the corresponding coded levels employed in the design.

**Table 4:** Range and levels of variables used for BBD.

Independent variables	Symbols	−1	0	+1
Static force, *F* (N)	X_1_	180	280	380
Vibration amplitudes, *A* (μm)	X_2_	6	11	16
Ball diameter, *D* (mm)	X_3_	4	6	8
Frequency, f (kHz)	X_4_	15	20	25

Table 5 displays the 27 results design of experiments which is obtained from proposed model.

**Table 5:** Results of the experiments.

Run	*F* (N)	*f* (kHz)	*A* (µm)	*D* (mm)	$\overset{R}{\underset{x}{\sigma }}$ (MPa)	z_0_ (µm)
**1**	180	15	11	6	−624	158.8
**2**	180	25	11	6	−668.6	177.8
**3**	380	15	11	6	−666.5	180
**4**	380	25	11	6	−711.6	199
**5**	180	20	6	6	−601.5	148.8
**6**	180	20	16	6	−684.2	184.2
**7**	380	20	6	6	−643.7	170
**8**	380	20	16	6	−727.4	205.4
**9**	180	20	11	4	−694.8	128.2
**10**	180	20	11	8	−623.5	207.9
**11**	380	20	11	4	−758	146.7
**12**	380	20	11	8	−656.2	231.3
**13**	280	15	6	6	−607	152.7
**14**	280	15	16	6	−679	184.3
**15**	280	25	6	6	−640	167.6
**16**	280	25	16	6	−733.4	206.3
**17**	280	15	11	4	−705.3	131.8
**18**	280	15	11	8	−617.8	207.4
**19**	280	25	11	4	−750.7	144.5
**20**	280	25	11	8	−662.4	232.7
**21**	280	20	6	4	−682.3	125.1
**22**	280	20	6	8	−595.5	194
**23**	280	20	16	4	−766.6	148.7
**24**	280	20	16	8	−678	241.3
**25**	280	20	11	6	−671	180.9
**26**	280	20	11	6	−671	180.9
**27**	280	20	11	6	−671	180.9

Equations (70) and (71) present the quadratic response surface models developed for the investigated output responses. The corresponding coefficients of determination (*R*²) indicate satisfactory agreement between the response surface approximations and the analytical data generated within the considered validation conditions.

It should be noted that the response surface models were constructed using data generated from the validated analytical framework rather than from independent experimental measurements. Consequently, the high R² values mainly reflect the internal consistency and approximation capability of the response surface models within the investigated parameter range and the scope of the present study, and should not be interpreted as evidence of universal predictive applicability. The developed response surfaces should therefore be regarded primarily as computationally efficient tools for parametric analysis and process optimization of ultrasonic rolling conditions.


$$\begin{array}{cc} \overset{R}{\underset{x}{\sigma }}= & -\text{608}.\text{619 }+(-\text{0}.\text{55725})F+(\text{53}.\text{1883})D+(-\text{8}.\text{34733})A+(-\text{4}.\text{221})f+(\text{0}.\text{038125})F*D\\ & +(-\text{0}.\text{0005})F*A+(-\text{0}.\text{00025})F*f+(\text{0}.\text{045})D*A+(\text{0}.\text{02})D*f+(-\text{0}.\text{214})A*f+(\text{0}.\text{000208333})F^{\text{2}}\\ & +(-\text{3}.\text{57604})D^{\text{2}}+(\text{0}.\text{190333})A^{\text{2}}+(\text{0}.\text{0518333})f^{\text{2}}(R\text{2}=\text{0}.\text{9996}),(\text{adj}=\text{0}.\text{9992}) \end{array}$$ (70)

$$\begin{array}{cc} Z_{\text{0}}= & \text{12}.\text{0007 }+(\text{0}.\text{141167})F+(\text{9}.\text{18})D+(\text{0}.\text{762})A+(\text{0}.\text{317333})f+(\text{0}.\text{006125})F*D\\ & \;\;+(-\text{4}.\text{82386e}-\text{17})F*A+(\text{7}.\text{22399e}-\text{17})F*f+(\text{0}.\text{5925})D*A+(\text{0}.\text{315})D*f\\ & \;\;+(\text{0}.\text{071})A*f+(-\text{0}.\text{000129167})F^{\text{2}}+(-\text{0}.\text{276042})D^{\text{2}}+(-\text{0}.\text{100167})A^{\text{2}}\\ & \;\;+(-\text{0}.\text{0276667})f^{\text{2}}(R\text{2}=\text{0}.\text{9999}),(\text{adj}=\text{0}.\text{9999}) \end{array}$$ (71)

#### 3.2.3. Parametric study.

**Fig 5:**
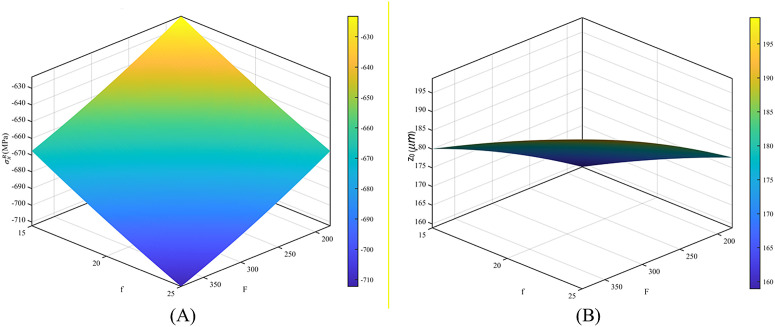
Effect of static force and frequency.

***Effect of static force and frequency:*** Figs 5A and Fig 5B depict the relationship between tangential residual stress $\overset{R}{\underset{x}{\sigma }}$and the maximum hardened layer depth *z*_0_ concerning static load *F* and ultrasonic frequency *f*, with ball diameter *D* and amplitude *A* maintained constant at 6 mm and 11 µm, respectively. It was seen that $\overset{R}{\underset{x}{\sigma }}$ increased monotonically with rising *F* and *f*, suggesting that both factors contributed to the formation of compressive residual stress.

The slope along the *F-*axis is steeper than the slope along the *f*-axis, indicating that the influence of static load was considerably stronger than that of ultrasonic frequency. When the static load *F* increased from 180 N to 380 N, the magnitude of compressive residual stress $\overset{R}{\underset{x}{\sigma }}$ increased by approximately 40 MPa. In contrast, increasing frequency from 15 kHz to 25 kHz produced only a modest increase of approximately 10 MPa. Increasing either parameter resulted in greater compressive stresses; however, the frequency primarily altered the homogeneity of the stress field rather than its intensity. In conclusion, it was demonstrated that the static load is the primary factor responsible for the emergence of surface compressive residual stress. The ultrasonic frequency has a small effect on this range. The static load exerted a significant influence on residual stress evolution, whereas the hardened layer depth z_0_ exhibited comparatively low sensitivity to the investigated process parameters. The response surface remains relatively planar, indicating that both parameters exert comparatively weak influence on z_0_ within the investigated range. At higher ultrasonic frequencies and moderate stress levels, the hardened layer depth z_0_ increases slightly. This indicates that the hardened layer depth is relatively insensitive to variations in these parameters within the investigated range.

**Fig 6:**
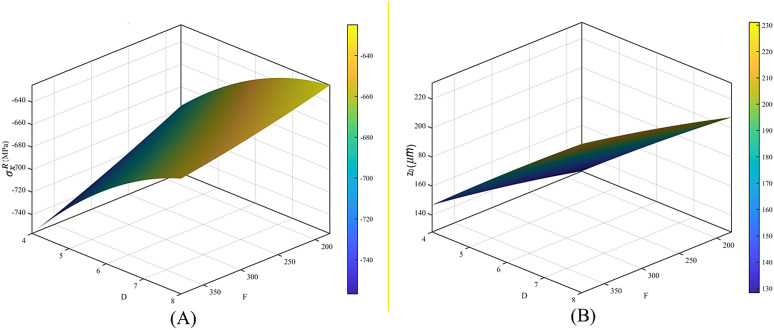
Effect of static force and ball diameter.

***Effect of static force and ball diameter:*** Fig 6 depicts the response surface of the tangential residual stress $\overset{R}{\underset{x}{\sigma }}$and the maximum hardened layer depth z_0_ as functions of the static load F and ball diameter D, with amplitude A and frequency f maintained at 11 µm and 20 kHz, respectively. The response surface indicates that the magnitude of compressive residual stress increases as ball diameter decreases, following a pronounced nonlinear trend as shown in Fig 6A. As the roller diameter decreases, the compressive residual stress increases, whereas the hardened layer depth gradually decreases as shown in Fig 6B

**Fig 7:**
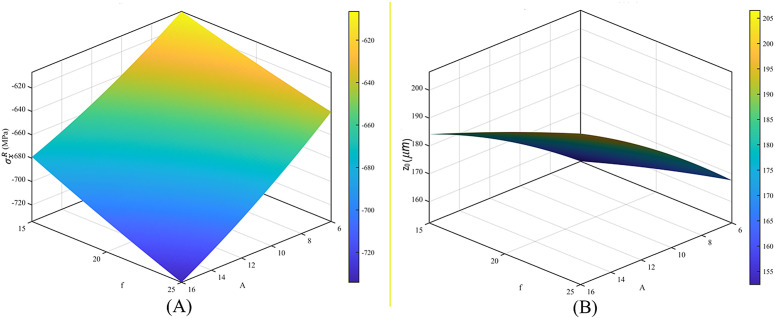
Effect of amplitude and frequency.

***Effects of amplitude and frequency:*** Fig 7A shows the response surface of residual stress $\overset{R}{\underset{x}{\sigma }}$ as a function of amplitude *A* and frequency *f*. The static load *F* is set at 280 N and the diameter of the ball D is set at 6 mm. The response surface indicates that the compressive residual stress gradually increases with vibration amplitude and ultrasonic frequency. This indicates that both parameters contribute positively to compressive residual stress development. However, the relatively smooth surface curvature and gentle gradient indicate moderate parameter sensitivity, with vibration amplitude exerting a slightly stronger influence than ultrasonic frequency. This behavior suggests that higher vibration amplitudes enhance cyclic plastic deformation and surface densification, whereas the influence of ultrasonic frequency remains comparatively less pronounced. Fig 7B shows the response surface of the hardened layer depth z_0_ when the conditions are the same. The variation in hardened layer depth z_0_ remains relatively limited over the investigated parameter range, showing a shallow surface curvature and a small range of 155–205 µm.

**Fig 8:**
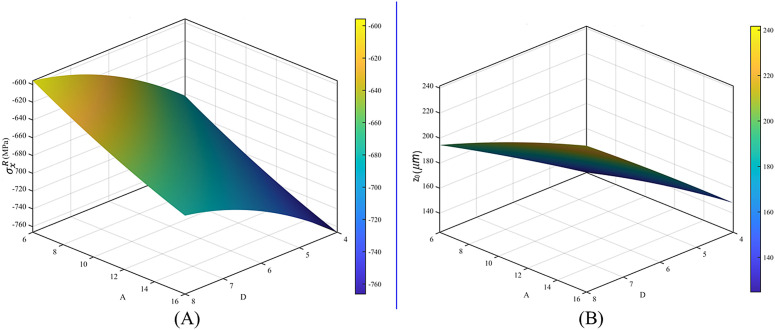
Effect of ball diameter and amplitude.

***Effect of ball diameter and amplitude:*** Fig 8A illustrates the variations in residual stress $\overset{R}{\underset{x}{\sigma }}$ as a function of vibration amplitude *A* and ball diameter *D* when the static load *F* is set at 280 N and the frequency *f* is set at 20 kHz. The response surface demonstrates that the magnitude of compressive residual stress increases as ball diameter decreases, whereas the influence of vibration amplitude is comparatively less pronounced. The curvature of the response surface indicates that ball diameter significantly influences compressive residual stress development, likely because smaller contact regions generate more concentrated subsurface plastic deformation. Fig 8B shows the response surface of the hardened layer depth $z_{\text{0}}$ as a function of amplitude *A* and ball diameter *D*, with the same fixed conditions. The response surface exhibits a mild and nearly linear trend. Increasing vibration amplitude slightly increases the hardened layer depth z_0_, whereas increasing ball diameter results in a slight reduction in the predicted hardened layer depth. This behavior suggests that higher ultrasonic amplitudes enhance surface hardening through increased cyclic vibrational energy transfer, whereas excessively large ball diameters may promote excessive plastic deformation, thereby limiting the effective penetration depth of the hardened layer.

**Fig 9:**
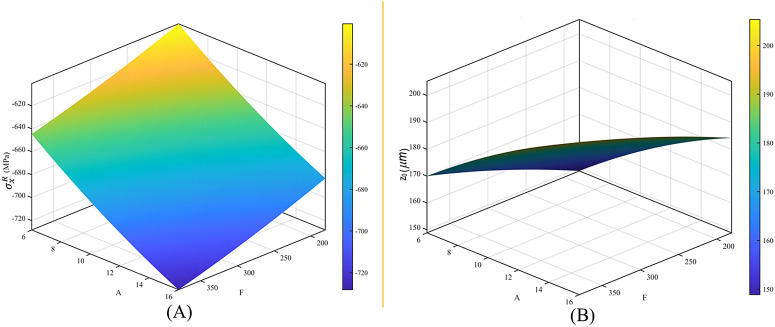
Effect of static force and amplitude.

***Effect of static force and amplitude:*** Fig 9A shows the response surface of residual stress $\overset{R}{\underset{x}{\sigma }}$ as a function of amplitude *A* and static force *F*. The ball diameter *D* and frequency *f* are kept at 6 *mm* and 20 *kHz*, respectively. The response surface indicates a continuous increase in compressive residual stress with increasing static load and vibration amplitude. Both parameters contribute positively to compressive residual stress development. The steeper slope along the *F*-axis shows that static force is the most important factor, while amplitude has a secondary but helpful effect. This behavior can be explained by the fact that higher contact pressure causes more plastic deformation and that higher vibration amplitudes introduce greater ultrasonic impact energy into the contact region. Fig 9B shows the response surface of the hardened layer depth *z*_0_ when the conditions are the same. The surface shows a small upward trend as *F* and *A* go up, which suggests that both parameters have a small positive effect on *z*_0_. The curvature is very small, which means that the change in hardened depth is still small (about 150–200 µm). This indicates that hardened layer depth is comparatively less sensitive to variations in amplitude and static force. The observed increases are likely associated with the combined effects of mechanical loading and vibrational energy, which enhance subsurface strengthening.

**Fig 10:**
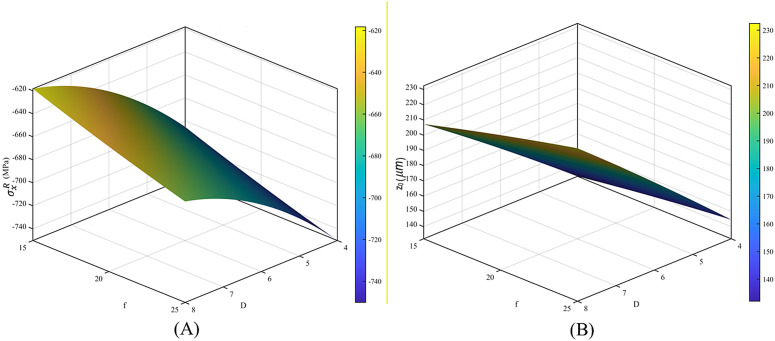
Effect of ball diameter and frequency.

***Effect of ball diameter and frequency:*** Fig 10A illustrates the response surface of residual stress $\overset{R}{\underset{x}{\sigma }}$ as a function of ball diameter *D* and frequency *f* while static load *F* and amplitude *A* are fixed at 280 *N*, 11 *μm*. The surface shows a pronounced increase in the magnitude of compressive residual stress as ball diameter decreases, whereas the effect of frequency remains comparatively small. Fig 10B presents the response surface of hardened layer depth *z*_0_ under the same fixed conditions. The response surface remains relatively planar, showing a mild decline in *z*_0_ with increasing ball diameter and a slight increase with frequency. The overall variation (150–230 µm) is modest, suggesting that both parameters exhibit comparatively weak influence on hardened layer depth. This behavior implies that while frequency may assist energy transfer during surface processing, excessively large ball diameters may induce excessive subsurface deformation, slightly reducing the effective hardening thickness.

***Discussion of Response Surface Behavior:*** The overall response surface analysis shows that the investigated process parameters affect residual stress evolution and hardened layer development through different but complementary mechanisms during ultrasonic surface rolling. Among the examined variables, static load *F* and ball diameter *D* exhibit the most pronounced influence on both compressive residual stress $\overset{R}{\underset{x}{\sigma }}$and hardened layer depth z_0_. This behavior can be attributed to their direct roles in governing Hertzian contact pressure, contact area, and the extent of subsurface plastic deformation.

Increasing the static load increases the mean contact pressure between the rolling ball and the Ti6Al4V surface, thereby promoting irreversible plastic strain accumulation beneath the treated surface. During unloading, the mismatch between the plastically deformed near-surface layer and the elastically constrained substrate contributes to the formation of compressive residual stress. Meanwhile, ball diameter affects the spatial distribution of contact pressure. A smaller ball diameter may increase local contact pressure and peak compressive stress near the surface, whereas a larger ball diameter distributes the contact load over a wider region and may promote a deeper plastically affected layer. Therefore, *F* and *D* strongly influence both the magnitude and depth characteristics of the residual stress field.

The influence of vibration amplitude *A* is mainly associated with the dynamic impact energy introduced by ultrasonic oscillation. Higher vibration amplitudes enhance the dynamic kinetic energy transferred to the contact region during each ultrasonic cycle, thereby intensifying cyclic plastic deformation, strain accumulation, and ultrasonic-assisted material flow. Although amplitude does not govern the mean Hertzian contact pressure as directly as static load and ball diameter, it contributes to residual stress stabilization and can enhance the development of the compressive stress layer through repeated dynamic loading.

Ultrasonic frequency *f* shows a comparatively weaker influence on peak compressive residual stress within the investigated parameter range. According to Eq. (16), the maximum vibration velocity of the tool is proportional to both ultrasonic frequency and vibration amplitude. Therefore, increasing frequency can enhance the rate of cyclic impact and ultrasonic energy transfer at the contact interface. This repeated high-frequency loading may promote cyclic plastic deformation, dislocation activity, and stress redistribution within the near-surface region. However, unlike static load and ball diameter, frequency does not directly govern the mean Hertzian contact pressure or the size of the primary contact zone. Consequently, within the investigated parameter range, its contribution to peak compressive residual stress formation remains less pronounced than the direct mechanical effects associated with static load and contact geometry.

Overall, the combined response surface analysis indicates that mechanical contact parameters, particularly static load and ball diameter, primarily govern the formation and spatial extent of the compressive residual stress field, whereas ultrasonic vibration parameters mainly enhance cyclic deformation efficiency and microstructural refinement. These findings provide a physically interpretable basis for optimizing ultrasonic rolling conditions to achieve a desirable balance between compressive residual stress magnitude and compressive layer depth.

## 4. Optimization

The surface compressive residual stress and hardened layer depth are two important indicators governing fatigue strengthening, crack resistance, and component service life during the ultrasonic surface rolling process. Higher and more uniformly distributed compressive residual stresses suppress crack initiation and propagation, whereas a deeper hardened layer enlarges the mechanically strengthened region and improves the long-term stability of the surface strengthening effect, particularly under cyclic loading and severe service conditions.

However, focusing exclusively on only one of these responses may reduce the overall effectiveness of the surface treatment. For example, high compressive residual stress associated with a shallow hardened layer may provide only short-term strengthening benefits, whereas a deeper layer accompanied by insufficient compressive stress may result in limited fatigue improvement. Therefore, simultaneous optimization of compressive residual stress and hardened layer depth is essential for maintaining surface integrity and long-term reliability while achieving an effective balance between mechanical performance, fatigue resistance, and process feasibility.

The following section presents the multi-parameter optimization results obtained to identify the optimal combination of static load *F*, ball diameter *D*, vibration amplitude *A*, and ultrasonic frequency *f* for maximizing compressive residual stress while maintaining an appropriate hardened layer depth.

The suggested multi-objective design optimization problem seeks to concurrently optimize both the residual outcome and the depth at which this outcome reaches its maximum as:


$$\begin{array}{c} \left\{ \begin{array}{c} \text{7}\text{0}\text{0}MPa\leq \overset{R}{\underset{x}{\sigma }}\leq \text{8}\text{0}\text{0}MPa\\ \text{1}\text{6}\text{0}\mu m\leq z_{\text{0}}\leq \text{2}\text{0}\text{0}\mu m\; \end{array}\right. \end{array}$$

Subject to the following constraints:


$$\begin{array}{c} \left\{ \begin{array}{c} \text{1}\text{8}\text{0}N\leq F\leq \text{3}\text{8}\text{0}N\\ \begin{array}{c} \text{1}\text{5}kHz\leq f\leq \text{2}\text{5}kHz\\ \text{4}mm\leq D\leq \text{8}mm\\ \text{6}\mu m\leq A\leq \text{1}\text{6}\mu m \end{array} \end{array}\right. \end{array}$$

This study employs a hybrid approach utilizing genetic algorithms (GA) and sequential quadratic programming (SQP) to tackle the optimization problem. As previously mentioned, SQP is a robust nonlinear mathematical programming method capable of accurately identifying a local optimal solution; nevertheless, it functions just as a local optimizer and lacks a mechanism for global solution exploration. In contrast, a notable and widely utilized stochastic global optimization method is the genetic algorithm (GA). To accurately get the global optimal solutions, the SQP solver utilizes the optimal solutions from GA that are proximate to the true global optimum as initial points in this hybrid optimization method. This method has demonstrated efficacy in the design optimization of multiphysics problems without an analytical assurance of a global solution.

The optimal combination of design variables, including static force, ball diameter, vibration amplitude, and frequency, has been established through the design optimization problem outlined in the previous section. The ideal residual stress parameters are as follows: *F* = 252.4 *N*, D = 6 *mm*, *A* = 15.78 *μm*, *f* = 20 *kHz*, with the predicted residual stress and hardened layer depth being: $\overset{R}{\underset{x}{\sigma }}$= −700 *MPa*, z_0_ = 192.483 *μm*.

## 5. Conclusion

This study developed a unified analytical framework for predicting residual stress evolution and hardened layer development during ultrasonic rolling of Ti6Al4V alloy. The proposed model incorporates the effects of static load, vibration amplitude, ultrasonic frequency, rolling ball material, and ball diameter through Hertzian contact mechanics and elastic–plastic deformation theory. The developed response surface models were systematically employed to investigate the influence of key process parameters on compressive residual stress distribution and hardened layer depth.

The analytical predictions demonstrated reasonable agreement with experimentally reported residual stress profiles, confirming the capability of the proposed framework to capture the dominant characteristics of residual stress evolution during ultrasonic rolling. The parametric analysis revealed that static load and rolling ball diameter predominantly govern the formation and spatial extent of the compressive residual stress field, whereas ultrasonic vibration parameters, particularly vibration amplitude and frequency, mainly contribute to enhancing cyclic deformation efficiency and microstructural homogeneity.

Furthermore, a multi-parameter optimization framework was established to simultaneously optimize compressive residual stress magnitude and hardened layer depth within the investigated parameter range. The obtained results provide a computationally efficient and physically interpretable basis for optimizing ultrasonic rolling conditions and improving the fatigue performance and long-term surface integrity of Ti6Al4V components.

## Supporting information

S1 Data Numerical data used for the validation curves presented in Fig 4. (XLSX)

S2 Data Detailed RMSE and MAE calculations used for the quantitative validation analysis. (XLSX)
