# Supplementary material for: An analytical method to optimize residual stress in the ultrasonic rolling of titanium alloys Ti6Al4V
Source: PLoS One. 2026 Jun 26;21(6):e0351986. doi: 10.1371/journal.pone.0351986 (PMC13308835; doi:10.1371/journal.pone.0351986)
Supplement: Attachment — Submitted filename: AnswerSheet_truyen_for_PLOSONE.docx [file pone.0351986.s004.docx]

**ANSWERS WITH RESPECT TO REFEREE REPORT**

**PLOS ONE**

**PONE-D-26-00227**

**An analytical method to optimize residual stress in the ultrasonic rolling of titanium alloys Ti6Al4V**

**Dear Editor and Reviewers,**

We would like to express our sincere gratitude to the Editor and the Reviewers for their constructive comments and valuable suggestions on our manuscript entitled " **An analytical method to optimize residual stress in the ultrasonic rolling of titanium alloys Ti6Al4V**" (Manuscript ID: **PONE-D-26-00227**).These comments are exceptionally helpful for improving the quality of our manuscript. We have carefully considered all comments and made corresponding revisions in the revised manuscript. All changes in the revised manuscript have been highlighted in red color / blue highlight for easy tracking.

Below is our point-by-point response to the reviewers’ comments.

Best regards,

Authors

**COMMENTS FOR AUTHORS & ANSWERS**

**Reviewer #1**: The manuscript describes a technically sound piece of scientific research with data that supports the conclusions. Experiments have been conducted rigorously. The conclusions are drawn appropriately based on the data presented.

**Additional Clarification on Validation Parameters**

The authors sincerely thank the reviewer for this constructive suggestion. In response, the validation section has been substantially expanded and strengthened in the revised manuscript.

***First, the authors would like to clarify a textual inconsistency identified in the original manuscript regarding the description of the experimental parameters reported by Bozdana. During preparation of the original manuscript, an earlier draft of the validation section had initially been prepared using parameters associated with a different reference study. Although the validation discussion was subsequently revised to use the experimental residual stress data reported by Bozdana, several parameter values in the descriptive sentence were inadvertently not updated accordingly***.

After careful re-examination of the validation procedure, it was confirmed that the actual analytical calculations and residual stress predictions were performed using the correct validation parameters corresponding to the experimental conditions reported by Bozdana, namely a static load of 380 N, a rolling ball diameter of 6 mm, and a vibration amplitude of 10.9 μm. Therefore, this issue was limited to an incorrect textual description in the manuscript and did not affect the analytical calculations, residual stress distributions, response surface analysis, optimization results, or the conclusions of the study. The validation description in the revised manuscript has been corrected accordingly to ensure consistency and clarity.

In addition to the original validation case corresponding to the 380 N ultrasonic deep cold rolling (UDCR) condition reported by Bozdana, an additional validation case corresponding to the 30 N UDCR condition has been incorporated. This additional comparison allows the proposed analytical framework to be evaluated under both low-force and high-force ultrasonic rolling conditions.

Furthermore, quantitative validation metrics including peak stress error, peak depth error, root mean square error (RMSE), and mean absolute error (MAE) were introduced to evaluate the agreement between the analytical predictions and the experimentally observed residual stress profiles. The experimental residual stress data were digitized from the published residual stress profiles using the WebPlotDigitizer software.

The revised results demonstrate that the proposed analytical framework provides reasonable agreement with the experimentally observed residual stress evolution behavior under both validation conditions. In particular, for the 380 N condition, the predicted peak compressive residual stress and corresponding penetration depth show errors of approximately 1.45% and 6.67%, respectively.

The validation section has been corrected in the updated manuscript.

**3.1 Material and processing parameters**

The dimensions of the Ti6Al4V specimen in this study were established according to the experimental setup outlined by Bozdana et al. [24]. The specimen is a cuboid with dimensions of 5 mm x 5 mm x 6 mm. The parameters employed in the ultrasonic surface rolling process for prediction of the residual stress distribution in the proposed analytical model were as follows: (a) an ultrasonic frequency of 20 kHz, a static load of 30 N, a rolling ball diameter of 6 mm, and a vibration amplitude of 8.72 μm; and (b) an ultrasonic frequency of 20 kHz, a static load of 380 N, a rolling ball diameter of 6 mm, and a vibration amplitude of 10.9 μm. These parameter values correspond to the experimental conditions reported by Bozdana et al. in their ultrasonic deep cold rolling study. The initial surface residual stress produced by the turning operation is −7.24 MPa.

……………………………………………………………………………………………..

**“****3.2 Results and discussion**

**3.2.1 Validation of the Model**

To validate the predictive capability of the proposed analytical framework for residual stress evolution during ultrasonic surface rolling, the analytical results were compared with the experimental residual stress distributions reported by Bozdana [24] for Ti6Al4V alloy subjected to ultrasonic deep cold rolling (UDCR). Two validation conditions corresponding to static loads of 30 N and 380 N were considered to evaluate the model performance under both low-force and high-force loading conditions.

Figure 4 presents the comparison between the analytically predicted residual stress distributions and the experimentally measured residual stress profiles reported in Ref. [24]. The parameters used for prediction were as follows: (a) an ultrasonic frequency of 20 kHz, a static load of 30 N, a rolling ball diameter of 6 mm, and a vibration amplitude of 8.72 μm; and (b) an ultrasonic frequency of 20 kHz, a static load of 380 N, a rolling ball diameter of 6 mm, and a vibration amplitude of 10.9 μm. These parameter values correspond to the experimental conditions reported by Bozdana [24].

|  |  |
| --- | --- |
| a) F = 30 N, f = 20 kHz, A = 8.72µm | b) F = 380 N, f = 20 kHz, A = 10.9 µm |

Fig. 4. Comparison results between predictive model and experiment

For both loading conditions, the proposed analytical framework reasonably reproduces the experimentally observed residual stress evolution beneath the treated surface. The predicted results successfully capture the formation of the near-surface compressive residual stress (CRS) region as well as the general depth-dependent stress evolution trend. In both the analytical predictions and the experimental observations, the compressive residual stress gradually decreases with increasing depth and eventually approaches a nearly neutral stress state within the bulk material.

To further evaluate the predictive capability of the proposed analytical framework, additional quantitative error metrics were introduced based on comparisons between the analytical predictions and the experimentally observed residual stress profiles. The experimental residual stress data were extracted from the published figures using the WebPlotDigitizer software.

Since the proposed analytical framework was primarily developed to predict the dominant characteristics of near-surface compressive residual stress evolution and compressive residual stress layer development, the quantitative error analysis was performed within the near-peak compressive residual stress region rather than at the immediate surface. The surface point at 0 μm was excluded from the quantitative error calculation because the largest local discrepancy occurred at the immediate surface region, where residual stress measurements are highly sensitive to experimental uncertainty, surface preparation conditions, data extraction accuracy, and local microstructural effects that are not fully represented in the simplified analytical formulation. Accordingly, the quantitative error analysis was conducted within the ranges of 80–100 μm for the 30 N condition and 100–400 μm for the 380 N condition, corresponding to the near-peak compressive residual stress region.

The root mean square error (RMSE) and mean absolute error (MAE) were calculated as follows:

RMSE

$$RMSE=\sqrt{\frac{1}{n}\sum_{i=1}^{n} \left( \sigma_{pred,i}-\sigma_{exp,i} \right)^{2}}$$

(65)

MAE

$$MAE=\frac{1}{n}\sum_{i=1}^{n} \left| \sigma_{pred,i}-\sigma_{exp,i} \right|$$

(66)

where $\sigma_{pred,i}$ and $\sigma_{exp,i}$ are the predicted and experimental residual stress values at the *i^th^* depth location, respectively, and *n* is the number of comparison points.

Table 3 summarizes the quantitative comparison results between the proposed analytical model and the experimentally observed residual stress profiles reported by Bozdana [24].

Table 3. Quantitative comparison between the proposed analytical model and the experimental residual stress profiles reported by Bozdana

| Validation case | Depth range used for error analysis (µm) | Peak compressive residual stress (Exp.) [MPa] | Peak compressive residual stress (Pred.) [MPa] | Peak stress error (%) | Peak depth (Exp.) [µm] | Peak depth (Pred.) [µm] | Depth error (%) | RMSE (MPa) | MAE (MPa) |
| --- | --- | --- | --- | --- | --- | --- | --- | --- | --- |
| UDCR 30 N | 80-100 | -490 | -490 | 0.00 | 80 | 80 | 0.00 | 7.78 | 5.50 |
| UDCR 380 N | 100-400 | -690 | -689 | 0.14 | 380 | 300 | 21.05 | 47.51 | 38.00 |

For the 380 N condition, the analytical prediction gives a peak compressive residual stress of approximately −689 MPa at a depth of about 300 μm, compared with the experimentally observed value of approximately −690 MPa at a depth close to 380 μm. The corresponding peak stress and peak depth errors are approximately 0.14% and 21.05%, respectively. The RMSE and MAE values within the investigated near-peak compressive residual stress region are 47.51 MPa and 38.00 MPa, respectively.

For the 30 N condition, the predicted peak compressive residual stress and corresponding peak depth show good agreement with the experimentally observed values, with both the peak stress error and peak depth error equal to 0.00%. The corresponding RMSE and MAE values within the investigated near-peak region are 7.78 MPa and 5.50 MPa, respectively.

The obtained quantitative comparison results indicate that the proposed analytical framework can reasonably reproduce the dominant characteristics of near-surface residual stress evolution and compressive layer development during ultrasonic rolling of Ti6Al4V alloy within the investigated parameter range.

The remaining discrepancies between the analytical predictions and the experimental profiles may be associated with the simplifying assumptions adopted in the analytical formulation, including isotropic hardening behavior, simplified representation of the coupled static–dynamic loading condition, omission of strain-rate-dependent constitutive effects, and uncertainties associated with extraction of experimental data from published figures.

The proposed analytical framework adopts several simplifying assumptions to maintain computational efficiency and analytical tractability, including isotropic hardening behavior, neglect of the Bauschinger effect, simplified representation of the coupled static–dynamic loading condition, and omission of strain-rate-dependent constitutive effects and thermal influences. Although these simplifications may affect prediction accuracy under highly nonlinear cyclic deformation conditions associated with ultrasonic rolling, the proposed model still demonstrates reasonable agreement with experimentally observed residual stress profiles and reasonably captures the dominant trends governing residual stress evolution during ultrasonic rolling. Although some discrepancies remain between the analytical predictions and the experimentally observed residual stress profiles, particularly near the immediate surface region, the obtained quantitative error metrics demonstrate that the proposed analytical framework can reasonably capture the dominant characteristics of compressive residual stress evolution and compressive layer development during ultrasonic rolling of Ti6Al4V alloy. The obtained RMSE and MAE values within the near-peak compressive residual stress region, together with the good agreement in peak compressive residual stress magnitude and penetration depth, indicate that the proposed analytical model provides reasonable predictive capability for parametric analysis, process optimization, and preliminary engineering design applications. Considering the computational efficiency and physical interpretability of the analytical framework compared with computationally intensive finite-element simulations, the obtained prediction accuracy is considered acceptable within the scope of the present study.”

Please review the revised manuscript

Best regards,

Authors

**Reviewer #2**: Reviewer Comments

The manuscript presents an analytical framework for predicting and optimizing residual stress during ultrasonic rolling of Ti6Al4V. The integration of Hertzian contact theory, elastic–plastic deformation modeling, and response surface methodology is relevant and addresses an important problem in surface engineering and fatigue enhancement of titanium alloys.

The study demonstrates reasonable agreement between predicted and experimental residual stress profiles, with the model capturing both the magnitude and depth of peak compressive stress. This indicates that the proposed analytical formulation has potential practical value. However, several important issues should be addressed before the manuscript can be considered for publication:

**Comment 1. Novelty and Contribution**
The manuscript does not clearly articulate what is fundamentally new compared to existing analytical and semi-empirical models. The combination of Hertzian contact theory and response surface methodology is well established. The authors should explicitly define the unique contribution of this work and distinguish it from prior studies.

**Response**

The authors sincerely thank the reviewer for this valuable comment. In response, the novelty and scientific contribution of the present study have been clarified and strengthened in the revised manuscript.

The revised manuscript now explicitly emphasizes that the novelty of the present work does not arise from the isolated use of Hertzian contact mechanics or response surface methodology individually, since these approaches are already well established in the literature. Instead, the main contribution lies in their integration into a unified physics-based analytical framework for predicting and optimizing residual stress evolution during ultrasonic rolling of Ti6Al4V alloy.

Additional discussion has been incorporated to clarify that most previous studies primarily relied on experimental observations or computationally intensive finite-element simulations within relatively limited parameter ranges. In contrast, the proposed framework provides a computationally efficient analytical methodology capable of simultaneously predicting both compressive residual stress magnitude and compressive layer depth under coupled static and ultrasonic loading conditions.

The revised manuscript further clarifies that the proposed approach systematically evaluates the coupled influence of static load, vibration amplitude, ultrasonic frequency, and rolling ball radius on residual stress evolution and hardened layer development through a combined analytical–statistical framework. Consequently, the proposed methodology provides not only physical interpretation of the underlying deformation mechanisms, but also a computationally efficient tool for parametric analysis and process optimization of ultrasonic rolling for Ti6Al4V alloy.

The abstract and introduction section has been updated in the revised manuscript.

**“Abstract**

Ultrasonic rolling is an effective surface enhancement technique for titanium alloys because it introduces beneficial compressive residual stresses that improve fatigue resistance and service durability. In many aerospace and high-performance engineering applications, both the magnitude and penetration depth of the compressive residual stress field play critical roles in determining long-term structural reliability and resistance to crack initiation and propagation. However, the complex elastic–plastic deformation behavior induced by coupled static and ultrasonic loading makes accurate prediction and simultaneous optimization of these characteristics challenging.

This study develops a unified analytical framework for simultaneously predicting and optimizing compressive residual stress magnitude and compressive layer depth during ultrasonic rolling of Ti6Al4V alloy. The proposed approach combines Hertzian contact mechanics, elastic–plastic deformation theory, and response surface methodology to systematically investigate the effects of static load, vibration amplitude, ultrasonic frequency, and rolling ball radius on residual stress evolution and hardened layer development.

The analytical predictions were validated through comparison with experimentally reported residual stress profiles under different ultrasonic rolling conditions. The validation results demonstrate reasonable agreement between the analytical predictions and experimental observations in terms of residual stress distribution, peak compressive stress magnitude, and penetration depth. The parametric analysis indicates that static load and vibration amplitude exert dominant influences on residual stress evolution and compressive layer formation. The proposed framework provides a computationally efficient and physically interpretable methodology for analyzing and optimizing ultrasonic rolling parameters for Ti6Al4V alloy.

*Keywords*: Ultrasonic rolling, residual stress, elastic-plastic deformation, Titanium alloys, Ti6Al4V

**1. Introduction**

Conventional techniques for enhancing surface strength, such as shot peening, laser shock peening, liquid jet peening, and low-plasticity burnishing, induce compressive residual stresses that improve the wear resistance, fatigue endurance, and ductility of titanium components. Nonetheless, conventional approaches have several drawbacks, including significant plastic deformation, superficial layer depth, limited processing efficiency, and imprecise processing trajectories [1]. Ultrasonic vibration-assisted rolling has recently emerged as an effective method for surface treatment that preserves advantageous compressive residual stresses and enhances surface integrity during roughing or semi-finishing [2].

Ultrasonic surface rolling technology (URT) has gained prominence in the automotive, aerospace, and energy sectors because of its ability to enhance performance and longevity of components [3, 4]. In the automotive sector, URT minimizes wear on engines, gearboxes, and shafts operating at high load and speed [5]. This technology is utilized in the aerospace sector to prevent the failure and deterioration of turbine blades, landing gear, and structural components exposed to high pressure and cyclic stresses. Ultrasonic surface treatment benefits power generation systems, oil and gas turbine rotors, pressure vessels, and pipelines in the energy sector by mitigating stress corrosion cracking, thermal fatigue, and cyclic stress. URT is deemed highly appropriate for high-performance, safety-critical engineering applications because of these characteristics. It enhances safety and dependability while reducing maintenance expenses and downtime [6, 7]. Residual stress is essential for preventing stress corrosion cracking, since it impedes crack initiation and bolsters material strength [8]. It directly affects the durability, robustness, and load-bearing ability of engineering components. To ensure long-term reliability, it is crucial to accurately measure and alleviate residual stress [9]. Multiple studies demonstrate that the ultrasonic rolling process (URP) substantially affects the integrity of the material's surface, along with its resistance to fatigue, corrosion, and wear. This is because URP enhances the strength and environmental resilience of the treated material through the induction of controlled compressive residual stress and the refinement of the surface grain structure. Ultrasonic rolling has demonstrated a considerable improvement in surface homogeneity, augmented deformation, and an increase in the thickness of the residual compressive stress layer. The combination of static pressure and ultrasonic vibration causes substantial plastic deformation, promoting the refinement of underlying grains and the production of a thick nanocrystalline surface layer. This technique enhances wear and corrosion resistance while augmenting fatigue performance by stabilizing surface compressive stress. Ultrasonic rolling yields a more homogeneous and finer surface morphology than traditional mechanical rolling or shot peening. This makes it an efficient technique for producing high-performance engineering alloys [1, 5, 7, 8].

In recent years, the Ti6Al4V titanium alloy has been extensively utilized in aerospace, biomedical, and high-performance mechanical applications owing to its superior strength-to-weight ratio, excellent corrosion resistance, and biocompatibility. Within these safety-critical sectors, mitigating surface defects and enhancing surface integrity are paramount, as they directly dictate the fatigue strength, long-term durability, and structural reliability of vital components subject to cyclic mechanical loading. However, Ti6Al4V has poor thermal conductivity and limited plasticity at ambient temperature, potentially resulting in machining inaccuracies, including micro-cracks and tensile residual stresses [11]. To address these constraints, rolling treatments—particularly surface rolling and ultrasonic surface rolling—have been devised to improve surface integrity and mechanical performance [12]. The combined effects of severe plastic deformation, strain hardening, and the introduction of residual compressive stress enhance the fatigue strength, wear resistance, and longevity of Ti6Al4V components by rolling [13]. The rolling process generates compressive contact force and tangential shear, resulting in significant plastic strain on the surface vicinity. This initiates dynamic recovery and recrystallization processes, resulting in ultrafine or nanocrystalline grains [14]. The increased density of dislocations enhances the material's hardness and reduces its susceptibility to fracture. The compressive stress field generated beneath the surface prevents the occurrence of fractures during load cycling [15]. The enhanced grain structure further augments tribological performance and corrosion resistance by stabilizing surface oxides and reducing surface roughness. Ultrasonic-assisted rolling employs high-frequency vibrations to improve plastic flow, diminish frictional heating, and augment the penetration depth of the compressive stress layer relative to conventional rolling methods. Ultrasonic rolling treatment significantly improves the surface integrity, fatigue resistance, and long-term durability of Ti6Al4V components, rendering it an invaluable post-processing technique for aerospace and biomedical applications [16].

During ultrasonic rolling, the cyclic plastic deformation process is highly complex due to the combined effects of static contact loading and high-frequency ultrasonic vibration. The rolling ball undergoes nonlinear dynamic interaction with the material surface, resulting in complex stress redistribution and residual compressive stress formation within the near-surface region. Experimental studies have shown that process parameters such as static load, vibration amplitude, ultrasonic frequency, and rolling ball diameter strongly influence the resulting residual stress distribution and hardened layer thickness [16–18]. Depending on the selected parameter combinations, these factors may produce either beneficial or unfavorable residual stress states.

In many aerospace and high-performance engineering applications, both the magnitude of compressive residual stress and the penetration depth of the compressive stress layer play critical roles in determining fatigue resistance and long-term structural reliability. For example, Ti6Al4V is widely used in turbine blades, landing gear components, aircraft fasteners, biomedical implants, and high-speed rotating shafts, where components are frequently subjected to cyclic loading and subsurface crack propagation. In such applications, a high compressive residual stress confined only to a shallow surface region may not provide sufficient resistance against crack initiation and crack growth under long-term service conditions. Therefore, simultaneous control of both compressive residual stress magnitude and compressive layer depth is essential for effective ultrasonic rolling process optimization and long-term fatigue performance enhancement.

Despite extensive experimental and numerical investigations on ultrasonic rolling of titanium alloys, most existing studies rely primarily on empirical observations or computationally expensive finite-element simulations within limited parameter ranges. Furthermore, previous research has mainly focused on qualitative trend analysis rather than systematic analytical prediction and optimization of residual stress characteristics. The cyclic and nonlinear nature of ultrasonic-assisted contact deformation also makes it difficult to establish generalized analytical relationships between process parameters and residual stress evolution.

To address these limitations, the present study develops a unified analytical framework for ultrasonic rolling of Ti6Al4V alloy based on Hertzian contact mechanics and elastic–plastic deformation theory. Unlike conventional empirical approaches, the proposed framework enables simultaneous analytical prediction of both compressive residual stress magnitude and compressive layer depth under coupled static and ultrasonic loading conditions. In addition, a Design of Experiments (DoE) approach combined with response surface methodology (RSM) is employed to systematically evaluate the effects of process parameters and their interaction effects on residual stress evolution.

The proposed methodology provides a computationally efficient and physically interpretable framework for analyzing and optimizing ultrasonic rolling parameters for Ti6Al4V alloy. The analytical predictions are further validated through comparison with experimentally reported residual stress profiles available in the literature.”

**Comment 2. Model Validation**

Validation is limited to comparison with a single experimental dataset from the literature. This is insufficient for establishing predictive robustness. Additional validation using independent datasets, or at minimum quantitative error metrics (e.g., RMSE, percentage error), should be included.

**Response**

The authors sincerely thank the reviewer for this constructive suggestion. In response, the validation section has been expanded and revised in the revised manuscript.

First, the authors would like to clarify a textual inconsistency identified in the original manuscript regarding the description of the experimental parameters reported by Bozdana. During preparation of the original manuscript, an earlier draft of the validation section had initially been prepared using parameters associated with a different reference study. Although the validation discussion was subsequently revised to use the experimental residual stress data reported by Bozdana, several parameter values in the descriptive sentence were inadvertently not updated accordingly.

After careful re-examination of the validation procedure, it was confirmed that the actual analytical calculations and residual stress predictions were performed using the correct validation parameters corresponding to the experimental conditions reported by Bozdana, namely a static load of 380 N, a rolling ball diameter of 6 mm, and a vibration amplitude of 10.9 μm. Therefore, this issue was limited to an incorrect textual description in the manuscript and did not affect the analytical calculations, residual stress distributions, response surface analysis, optimization results, or the conclusions of the study. The validation description in the revised manuscript has been corrected accordingly to ensure consistency and clarity.

In addition to the original validation case corresponding to the 380 N ultrasonic deep cold rolling (UDCR) condition reported by Bozdana, an additional validation case corresponding to the 30 N UDCR condition has been incorporated. This additional comparison allows the proposed analytical framework to be evaluated under both low-force and high-force ultrasonic rolling conditions.

Furthermore, quantitative validation metrics including peak stress error, peak depth error, root mean square error (RMSE), and mean absolute error (MAE) were introduced to evaluate the agreement between the analytical predictions and the experimentally observed residual stress profiles. The experimental residual stress data were digitized from the published residual stress profiles using the WebPlotDigitizer software.

The revised results demonstrate that the proposed analytical framework provides reasonable agreement with the experimentally observed residual stress evolution behavior under both validation conditions. In particular, for the 380 N condition, the predicted peak compressive residual stress and corresponding penetration depth show errors of approximately 1.45% and 6.67%, respectively.

The validation section has been expanded and revised in the updated manuscript.

**“3.1 Material and processing parameters**

The dimensions of the Ti6Al4V specimen in this study were established according to the experimental setup outlined by Bozdana et al. [24]. The specimen is a cuboid with dimensions of 5 mm x 5 mm x 6 mm. The parameters employed in the ultrasonic surface rolling process for prediction of the residual stress distribution in the proposed analytical model were as follows: (a) an ultrasonic frequency of 20 kHz, a static load of 30 N, a rolling ball diameter of 6 mm, and a vibration amplitude of 8.72 μm; and (b) an ultrasonic frequency of 20 kHz, a static load of 380 N, a rolling ball diameter of 6 mm, and a vibration amplitude of 10.9 μm. These parameter values correspond to the experimental conditions reported by Bozdana et al. in their ultrasonic deep cold rolling study. The initial surface residual stress produced by the turning operation is −7.24 MPa.

**………………………………………………………………………………………………**

**3.2 Results and discussion**

**3.2.1 Validation of the Model**

To validate the predictive capability of the proposed analytical framework for residual stress evolution during ultrasonic surface rolling, the analytical results were compared with the experimental residual stress distributions reported by Bozdana [24] for Ti6Al4V alloy subjected to ultrasonic deep cold rolling (UDCR). Two validation conditions corresponding to static loads of 30 N and 380 N were considered to evaluate the model performance under both low-force and high-force loading conditions.

Figure 4 presents the comparison between the analytically predicted residual stress distributions and the experimentally measured residual stress profiles reported in Ref. [24]. The parameters used for prediction were as follows: (a) an ultrasonic frequency of 20 kHz, a static load of 30 N, a rolling ball diameter of 6 mm, and a vibration amplitude of 8.72 μm; and (b) an ultrasonic frequency of 20 kHz, a static load of 380 N, a rolling ball diameter of 6 mm, and a vibration amplitude of 10.9 μm. These parameter values correspond to the experimental conditions reported by Bozdana [24].

|  |  |
| --- | --- |
| a) F = 30 N, f = 20 kHz, A = 8.72µm | b) F = 380 N, f = 20 kHz, A = 10.9 µm |

Fig. 4. Comparison results between predictive model and experiment

For both loading conditions, the proposed analytical framework reasonably reproduces the experimentally observed residual stress evolution beneath the treated surface. The predicted results successfully capture the formation of the near-surface compressive residual stress (CRS) region as well as the general depth-dependent stress evolution trend. In both the analytical predictions and the experimental observations, the compressive residual stress gradually decreases with increasing depth and eventually approaches a nearly neutral stress state within the bulk material.

To further evaluate the predictive capability of the proposed analytical framework, additional quantitative error metrics were introduced based on comparisons between the analytical predictions and the experimentally observed residual stress profiles. The experimental residual stress data were extracted from the published figures using the WebPlotDigitizer software.

Since the proposed analytical framework was primarily developed to predict the dominant characteristics of near-surface compressive residual stress evolution and compressive residual stress layer development, the quantitative error analysis was performed within the near-peak compressive residual stress region rather than at the immediate surface. The surface point at 0 μm was excluded from the quantitative error calculation because the largest local discrepancy occurred at the immediate surface region, where residual stress measurements are highly sensitive to experimental uncertainty, surface preparation conditions, data extraction accuracy, and local microstructural effects that are not fully represented in the simplified analytical formulation. Accordingly, the quantitative error analysis was conducted within the ranges of 80–100 μm for the 30 N condition and 100–400 μm for the 380 N condition, corresponding to the near-peak compressive residual stress region.

The root mean square error (RMSE) and mean absolute error (MAE) were calculated as follows:

RMSE

$$RMSE=\sqrt{\frac{1}{n}\sum_{i=1}^{n} \left( \sigma_{pred,i}-\sigma_{exp,i} \right)^{2}}$$

(65)

MAE

$$MAE=\frac{1}{n}\sum_{i=1}^{n} \left| \sigma_{pred,i}-\sigma_{exp,i} \right|$$

(66)

where $\sigma_{pred,i}$ and $\sigma_{exp,i}$ are the predicted and experimental residual stress values at the *i^th^* depth location, respectively, and *n* is the number of comparison points.

Table 3 summarizes the quantitative comparison results between the proposed analytical model and the experimentally observed residual stress profiles reported by Bozdana [24].

Table 3. Quantitative comparison between the proposed analytical model and the experimental residual stress profiles reported by Bozdana

| Validation case | Depth range used for error analysis (µm) | Peak compressive residual stress (Exp.) [MPa] | Peak compressive residual stress (Pred.) [MPa] | Peak stress error (%) | Peak depth (Exp.) [µm] | Peak depth (Pred.) [µm] | Depth error (%) | RMSE (MPa) | MAE (MPa) |
| --- | --- | --- | --- | --- | --- | --- | --- | --- | --- |
| UDCR 30 N | 80-100 | -490 | -490 | 0.00 | 80 | 80 | 0.00 | 7.78 | 5.50 |
| UDCR 380 N | 100-400 | -690 | -689 | 0.14 | 380 | 300 | 21.05 | 47.51 | 38.00 |

For the 380 N condition, the analytical prediction gives a peak compressive residual stress of approximately −689 MPa at a depth of about 300 μm, compared with the experimentally observed value of approximately −690 MPa at a depth close to 380 μm. The corresponding peak stress and peak depth errors are approximately 0.14% and 21.05%, respectively. The RMSE and MAE values within the investigated near-peak compressive residual stress region are 47.51 MPa and 38.00 MPa, respectively.

For the 30 N condition, the predicted peak compressive residual stress and corresponding peak depth show good agreement with the experimentally observed values, with both the peak stress error and peak depth error equal to 0.00%. The corresponding RMSE and MAE values within the investigated near-peak region are 7.78 MPa and 5.50 MPa, respectively.

The obtained quantitative comparison results indicate that the proposed analytical framework can reasonably reproduce the dominant characteristics of near-surface residual stress evolution and compressive layer development during ultrasonic rolling of Ti6Al4V alloy within the investigated parameter range.

The remaining discrepancies between the analytical predictions and the experimental profiles may be associated with the simplifying assumptions adopted in the analytical formulation, including isotropic hardening behavior, simplified representation of the coupled static–dynamic loading condition, omission of strain-rate-dependent constitutive effects, and uncertainties associated with extraction of experimental data from published figures.

The proposed analytical framework adopts several simplifying assumptions to maintain computational efficiency and analytical tractability, including isotropic hardening behavior, neglect of the Bauschinger effect, simplified representation of the coupled static–dynamic loading condition, and omission of strain-rate-dependent constitutive effects and thermal influences. Although these simplifications may affect prediction accuracy under highly nonlinear cyclic deformation conditions associated with ultrasonic rolling, the proposed model still demonstrates reasonable agreement with experimentally observed residual stress profiles and reasonably captures the dominant trends governing residual stress evolution during ultrasonic rolling.

Although some discrepancies remain between the analytical predictions and the experimentally observed residual stress profiles, particularly near the immediate surface region, the obtained quantitative error metrics demonstrate that the proposed analytical framework can reasonably capture the dominant characteristics of compressive residual stress evolution and compressive layer development during ultrasonic rolling of Ti6Al4V alloy. The obtained RMSE and MAE values within the near-peak compressive residual stress region, together with the good agreement in peak compressive residual stress magnitude and penetration depth, indicate that the proposed analytical model provides reasonable predictive capability for parametric analysis, process optimization, and preliminary engineering design applications. Considering the computational efficiency and physical interpretability of the analytical framework compared with computationally intensive finite-element simulations, the obtained prediction accuracy is considered acceptable within the scope of the present study.”

**Comment 3. Statistical Modeling Concerns**

The response surface models are constructed using data generated from the analytical model itself rather than independent experimental data. This approach can lead to artificially high R² values (reported >0.99) and does not demonstrate true predictive capability. The authors should clarify this limitation and temper claims accordingly.

**Response**

The authors sincerely thank the reviewer for this important observation. In response, the revised manuscript has been updated to explicitly clarify the limitation associated with the response surface methodology used in the present study.

The revised discussion now clarifies that the response surface models were constructed using data generated from the validated analytical framework rather than from independent experimental measurements. Consequently, the high R² values should primarily be interpreted as indicators of internal consistency and surrogate approximation capability within the investigated parameter domain, rather than generalized predictive capability.

To avoid overstatement, the discussion and conclusions have been revised accordingly. The revised manuscript now emphasizes that the response surface models are intended mainly as computationally efficient tools for parametric analysis and process optimization.

**The revised manuscript has been updated accordingly.**

“Equations (70) and (71) present the quadratic response surface models developed for the investigated output responses. The corresponding coefficients of determination (*R*²) indicate satisfactory agreement between the response surface approximations and the analytical data generated within the investigated parameter range.

It should be noted that the response surface models were constructed using data generated from the validated analytical framework rather than from independent experimental measurements. Consequently, the high R² values mainly reflect the internal consistency and approximation capability of the response surface models within the investigated parameter range and the scope of the present study, and should not be interpreted as evidence of universal predictive applicability. The developed response surfaces should therefore be regarded primarily as computationally efficient tools for parametric analysis and process optimization of ultrasonic rolling conditions.”

**Comment 4. Model Assumptions**

Several simplifying assumptions are made, including isotropic hardening, neglect of the Bauschinger effect, and simplified superposition of static and dynamic loads. These assumptions may not fully capture the cyclic and high strain-rate nature of ultrasonic rolling. A discussion on the limitations and potential impact of these assumptions is needed.

**Response**

The authors sincerely thank the reviewer for this insightful comment. In response, an additional discussion regarding the assumptions and limitations of the proposed analytical framework has been incorporated into the revised manuscript.

The revised manuscript now explicitly acknowledges that several simplifying assumptions were adopted to maintain analytical tractability and computational efficiency, including isotropic hardening behavior, neglect of the Bauschinger effect, simplified representation of coupled static and ultrasonic dynamic loading, and omission of strain-rate-dependent constitutive effects and thermal influences.

The revised discussion further clarifies that these simplifications may influence prediction accuracy under highly nonlinear cyclic deformation conditions associated with ultrasonic rolling. Nevertheless, despite these assumptions, the proposed analytical framework still demonstrates reasonable agreement with experimentally observed residual stress profiles and successfully captures the dominant trends governing residual stress evolution.

**These limitations were added to the validation section 3.2.1 to clarify the assumptions and constraints associated with the proposed analytical framework.**

“The proposed analytical framework adopts several simplifying assumptions to maintain computational efficiency and analytical tractability, including isotropic hardening behavior, neglect of the Bauschinger effect, simplified representation of the coupled static–dynamic loading condition, and omission of strain-rate-dependent constitutive effects and thermal influences. Although these simplifications may affect prediction accuracy under highly nonlinear cyclic deformation conditions associated with ultrasonic rolling, the proposed model still demonstrates reasonable agreement with experimentally observed residual stress profiles and reasonably captures the dominant trends governing residual stress evolution during ultrasonic rolling.”

**Comment 5. Physical Interpretation of Results**

While parametric trends are presented, the physical explanations remain limited. The authors should provide deeper mechanistic insight into why certain parameters (e.g., static load vs frequency) dominate residual stress formation.

**Response**

The authors sincerely thank the reviewer for this valuable comment. In response, the discussion of the parametric study has been substantially expanded in the revised manuscript to provide deeper physical interpretation of the observed trends.

Additional mechanistic explanations have been incorporated regarding the effects of static load, ball diameter, vibration amplitude, and ultrasonic frequency on Hertzian contact pressure, cyclic plastic deformation, strain accumulation, and ultrasonic energy transfer during ultrasonic rolling.

The revised discussion further explains static load strongly influences the formation of the subsurface plastic deformation zone because it directly governs the Hertzian contact pressure. In contrast, vibration amplitude mainly enhances cyclic deformation efficiency and ultrasonic energy transfer through repeated dynamic impacts and acoustoplastic effects. The revised discussion also addresses the possibility of plastic deformation saturation at higher static loading conditions, which may increase the relative influence of vibration amplitude during multi-objective optimization.

This additional discussion has been incorporated into the discussion section of the revised manuscript.

“***Discussion of Response Surface Behavior***

The overall response surface analysis shows that the investigated process parameters affect residual stress evolution and hardened layer development through different but complementary mechanisms during ultrasonic surface rolling. Among the examined variables, static load *F* and ball diameter *D* exhibit the most pronounced influence on both compressive residual stress $\sigma_{x}^{R}$and hardened layer depth z_0_. This behavior can be attributed to their direct roles in governing Hertzian contact pressure, contact area, and the extent of subsurface plastic deformation.

Increasing the static load increases the mean contact pressure between the rolling ball and the Ti6Al4V surface, thereby promoting irreversible plastic strain accumulation beneath the treated surface. During unloading, the mismatch between the plastically deformed near-surface layer and the elastically constrained substrate contributes to the formation of compressive residual stress. Meanwhile, ball diameter affects the spatial distribution of contact pressure. A smaller ball diameter may increase local contact pressure and peak compressive stress near the surface, whereas a larger ball diameter distributes the contact load over a wider region and may promote a deeper plastically affected layer. Therefore, *F* and *D* strongly influence both the magnitude and depth characteristics of the residual stress field.

The influence of vibration amplitude *A* is mainly associated with the dynamic impact energy introduced by ultrasonic oscillation. Higher vibration amplitudes enhance the dynamic kinetic energy transferred to the contact region during each ultrasonic cycle, thereby intensifying cyclic plastic deformation, strain accumulation, and ultrasonic-assisted material flow. Although amplitude does not govern the mean Hertzian contact pressure as directly as static load and ball diameter, it contributes to residual stress stabilization and can enhance the development of the compressive stress layer through repeated dynamic loading.

Ultrasonic frequency *f* shows a comparatively weaker influence on peak compressive residual stress within the investigated parameter range. According to Eq. (16), the maximum vibration velocity of the tool is proportional to both ultrasonic frequency and vibration amplitude. Therefore, increasing frequency can enhance the rate of cyclic impact and ultrasonic energy transfer at the contact interface. This repeated high-frequency loading may promote cyclic plastic deformation, dislocation activity, and stress redistribution within the near-surface region. However, unlike static load and ball diameter, frequency does not directly govern the mean Hertzian contact pressure or the size of the primary contact zone. Consequently, within the investigated parameter range, its contribution to peak compressive residual stress formation remains less pronounced than the direct mechanical effects associated with static load and contact geometry.

Overall, the combined response surface analysis indicates that mechanical contact parameters, particularly static load and ball diameter, primarily govern the formation and spatial extent of the compressive residual stress field, whereas ultrasonic vibration parameters mainly enhance cyclic deformation efficiency and microstructural refinement. These findings provide a physically interpretable basis for optimizing ultrasonic rolling conditions to achieve a desirable balance between compressive residual stress magnitude and compressive layer depth.”

**6. Clarity and Language**

The manuscript requires significant language editing. There are grammatical errors, repetitive phrasing, and sections where clarity is reduced. Improving readability will strengthen the paper considerably.

**Response**

The authors sincerely thank the reviewer for this helpful comment. In response, the manuscript has undergone extensive language revision to improve clarity, readability, consistency of terminology, and overall academic writing quality.

Grammatical issues, repetitive phrasing, informal wording, and typographical errors throughout the manuscript were carefully corrected. In addition, several sections of the Introduction, Results and Discussion, and Conclusion were revised to improve scientific flow and clarity of presentation.

Particular attention was given to reducing repetitive sentence structures, improving transitions between paragraphs, and refining the physical interpretation of the analytical results. Terminology related to compressive residual stress and hardened layer depth was also standardized throughout the manuscript to improve consistency.

**All revised text has been highlighted in red in the revised manuscript.**

The authors sincerely appreciate the reviewer’s valuable comments. The revised manuscript has been carefully improved in terms of validation, clarification of novelty, and depth of analysis in accordance with the reviewer’s suggestions.

Best regards,

Authors
