# Supplementary material for: An analytical method to optimize residual stress in the ultrasonic rolling of titanium alloys Ti6Al4V
Source: PLoS One. 2026 Jun 26;21(6):e0351986. doi: 10.1371/journal.pone.0351986 (PMC13308835; doi:10.1371/journal.pone.0351986)
Supplement: Peer review history

# Peer review: Decision Letter 0

**DOI:** 10.1371/journal.pone.0351986.r001

**Authors:** Yashwanth Nanda Kumar

19 May 2026

PONE-D-26-00227

An analytical method to optimize residual stress in the ultrasonic rolling of titanium alloys Ti6Al4V

PLOS One

Dear Dr. le,

Thank you for submitting your manuscript to PLOS ONE. After careful consideration, we feel that it has merit but does not fully meet PLOS ONE’s publication criteria as it currently stands. Therefore, we invite you to submit a revised version of the manuscript that addresses the points raised during the review process.

Please submit your revised manuscript by Jul 03 2026 11:59PM. If you will need more time than this to complete your revisions, please reply to this message or contact the journal office at plosone@plos.org. When you're ready to submit your revision, log on to https://www.editorialmanager.com/pone/ and select the 'Submissions Needing Revision' folder to locate your manuscript file.

Please include the following items when submitting your revised manuscript:

- A letter that responds to each point raised by the academic editor and reviewer(s). You should upload this letter as a separate file labeled 'Response to Reviewers'.
- A marked-up copy of your manuscript that highlights changes made to the original version. You should upload this as a separate file labeled 'Revised Manuscript with Track Changes'.
- An unmarked version of your revised paper without tracked changes. You should upload this as a separate file labeled 'Manuscript'.

If you would like to make changes to your financial disclosure, please include your updated statement in your cover letter. Guidelines for resubmitting your figure files are available below the reviewer comments at the end of this letter.

If applicable, we recommend that you deposit your laboratory protocols in protocols.io to enhance the reproducibility of your results. Protocols.io assigns your protocol its own identifier (DOI) so that it can be cited independently in the future. For instructions see: https://journals.plos.org/plosone/s/submission-guidelines#loc-laboratory-protocols. Additionally, PLOS ONE offers an option for publishing peer-reviewed Lab Protocol articles, which describe protocols hosted on protocols.io. Read more information on sharing protocols at https://plos.org/protocols?utm_medium=editorial-email&utm_source=authorletters&utm_campaign=protocols.

As the corresponding author, your ORCID iD is verified in the submission system and will appear in the published article. PLOS supports the use of ORCID, and we encourage all coauthors to register for an ORCID iD and use it as well. Please encourage your coauthors to verify their ORCID iD within the submission system before final acceptance, as unverified ORCID iDs will not appear in the published article. *Only*  the individual author can complete the verification step; PLOS staff *cannot*  verify ORCID iDs on behalf of authors.

We look forward to receiving your revised manuscript.

Kind regards,

Yashwanth Nanda Kumar, PhD

Academic Editor

PLOS One

**Journal Requirements:**

When submitting your revision, we need you to address these additional requirements.

1. Please ensure that your manuscript meets PLOS ONE's style requirements, including those for file naming. The PLOS ONE style templates can be found at

https://journals.plos.org/plosone/s/file?id=wjVg/PLOSOne_formatting_sample_main_body.pdf and

https://journals.plos.org/plosone/s/file?id=ba62/PLOSOne_formatting_sample_title_authors_affiliations.pdf

2. Please note that PLOS One has specific guidelines on code sharing for submissions in which author-generated code underpins the findings in the manuscript. In these cases, we expect all author-generated code to be made available without restrictions upon publication of the work. Please review our guidelines at https://journals.plos.org/plosone/s/materials-and-software-sharing#loc-sharing-code and ensure that your code is shared in a way that follows best practice and facilitates reproducibility and reuse.

3. We note that your Data Availability Statement is currently as follows:

“All relevant data are within the manuscript and its Supporting Information files.”

Please confirm at this time whether or not your submission contains all raw data required to replicate the results of your study. Authors must share the “minimal data set” for their submission. PLOS defines the minimal data set to consist of the data required to replicate all study findings reported in the article, as well as related metadata and methods (https://journals.plos.org/plosone/s/data-availability#loc-minimal-data-set-definition).

For example, authors should submit the following data:

- The values behind the means, standard deviations and other measures reported;

- The values used to build graphs;

- The points extracted from images for analysis.

Authors do not need to submit their entire data set if only a portion of the data was used in the reported study.

If your submission does not contain these data, please either upload them as Supporting Information files or deposit them to a stable, public repository and provide us with the relevant URLs, DOIs, or accession numbers. For a list of recommended repositories, please see https://journals.plos.org/plosone/s/recommended-repositories.

If there are ethical or legal restrictions on sharing a de-identified data set, please explain them in detail (e.g., data contain potentially sensitive information, data are owned by a third-party organization, etc.) and who has imposed them (e.g., an ethics committee). Please also provide contact information for a data access committee, ethics committee, or other institutional body to which data requests may be sent. If data are owned by a third party, please indicate how others may request data access.

4. PLOS requires an ORCID iD for the corresponding author in Editorial Manager on papers submitted after December 6th, 2016. Please ensure that you have an ORCID iD and that it is validated in Editorial Manager. To do this, go to ‘Update my Information’ (in the upper left-hand corner of the main menu), and click on the Fetch/Validate link next to the ORCID field. This will take you to the ORCID site and allow you to create a new iD or authenticate a pre-existing iD in Editorial Manager.

5. If the reviewer comments include a recommendation to cite specific previously published works, please review and evaluate these publications to determine whether they are relevant and should be cited. There is no requirement to cite these works unless the editor has indicated otherwise.

Reviewers' comments:

Reviewer's Responses to Questions

**Comments to the Author**

1. Is the manuscript technically sound, and do the data support the conclusions?

The manuscript must describe a technically sound piece of scientific research with data that supports the conclusions. Experiments must have been conducted rigorously, with appropriate controls, replication, and sample sizes. The conclusions must be drawn appropriately based on the data presented.

Reviewer #1: Yes

Reviewer #2: Partly

**********

2. Has the statistical analysis been performed appropriately and rigorously?

Reviewer #1: Yes

Reviewer #2: No

**********

3. Have the authors made all data underlying the findings in their manuscript fully available?

The PLOS Data policy requires authors to make all data underlying the findings described in their manuscript fully available without restriction, with rare exception (please refer to the Data Availability Statement in the manuscript PDF file). The data should be provided as part of the manuscript or its supporting information, or deposited to a public repository. For example, in addition to summary statistics, the data points behind means, medians and variance measures should be available. If there are restrictions on publicly sharing data—e.g. participant privacy or use of data from a third party—those must be specified.

Reviewer #1: Yes

Reviewer #2: Yes

**********

4. Is the manuscript presented in an intelligible fashion and written in standard English?

PLOS ONE does not copyedit accepted manuscripts, so the language in submitted articles must be clear, correct, and unambiguous. Any typographical or grammatical errors should be corrected at revision, so please note any specific errors here.

Reviewer #1: Yes

Reviewer #2: No

**********

5. Review Comments to the Author

Please use the space provided to explain your answers to the questions above. You may also include additional comments for the author, including concerns about dual publication, research ethics, or publication ethics. (Please upload your review as an attachment if it exceeds 20,000 characters)

Reviewer #1: The manuscript describe a technically sound piece of scientific research with data that supports the conclusions. Experiments have been conducted rigorously. The conclusions are drawn appropriately based on the data presented.

Reviewer #2: Reviewer Comments

The manuscript presents an analytical framework for predicting and optimizing residual stress during ultrasonic rolling of Ti6Al4V. The integration of Hertzian contact theory, elastic–plastic deformation modeling, and response surface methodology is relevant and addresses an important problem in surface engineering and fatigue enhancement of titanium alloys.

The study demonstrates reasonable agreement between predicted and experimental residual stress profiles, with the model capturing both the magnitude and depth of peak compressive stress. This indicates that the proposed analytical formulation has potential practical value.

However, several important issues should be addressed before the manuscript can be considered for publication:

1. Novelty and Contribution

The manuscript does not clearly articulate what is fundamentally new compared to existing analytical and semi-empirical models. The combination of Hertzian contact theory and response surface methodology is well established. The authors should explicitly define the unique contribution of this work and distinguish it from prior studies.

2. Model Validation

Validation is limited to comparison with a single experimental dataset from the literature. This is insufficient for establishing predictive robustness. Additional validation using independent datasets, or at minimum quantitative error metrics (e.g., RMSE, percentage error), should be included.

3. Statistical Modeling Concerns

The response surface models are constructed using data generated from the analytical model itself rather than independent experimental data. This approach can lead to artificially high R² values (reported >0.99) and does not demonstrate true predictive capability. The authors should clarify this limitation and temper claims accordingly.

4. Model Assumptions

Several simplifying assumptions are made, including isotropic hardening, neglect of the Bauschinger effect, and simplified superposition of static and dynamic loads. These assumptions may not fully capture the cyclic and high strain-rate nature of ultrasonic rolling. A discussion on the limitations and potential impact of these assumptions is needed.

5. Physical Interpretation of Results

While parametric trends are presented, the physical explanations remain limited. The authors should provide deeper mechanistic insight into why certain parameters (e.g., static load vs frequency) dominate residual stress formation.

6. Clarity and Language

The manuscript requires significant language editing. There are grammatical errors, repetitive phrasing, and sections where clarity is reduced. Improving readability will strengthen the paper considerably.

Conclusion

The study addresses a relevant engineering problem and shows promising results, but requires major revision, particularly in terms of validation, clarity of contribution, and depth of analysis.

**********

6. PLOS authors have the option to publish the peer review history of their article (what does this mean?). If published, this will include your full peer review and any attached files.

If you choose “no”, your identity will remain anonymous but your review may still be made public.

**Do you want your identity to be public for this peer review?**  For information about this choice, including consent withdrawal, please see our Privacy Policy.

Reviewer #1: **Yes:** Arunangsu Das, Ph.D.

Reviewer #2: **Yes:** Uwais Al Qarn Ahmed

**********

To ensure your figures meet our technical requirements, please review our figure guidelines: https://journals.plos.org/plosone/s/figures

You may also use PLOS’s free figure tool, NAAS, to help you prepare publication quality figures: https://journals.plos.org/plosone/s/figures#loc-tools-for-figure-preparation.

NAAS will assess whether your figures meet our technical requirements by comparing each figure against our figure specifications.

# Author comment: Author response to Decision Letter 1

**DOI:** 10.1371/journal.pone.0351986.r002

27 May 2026

ANSWERS WITH RESPECT TO REFEREE REPORT

PLOS ONE

PONE-D-26-00227

An analytical method to optimize residual stress in the ultrasonic rolling of titanium alloys Ti6Al4V

Dear Editor and Reviewers,

We would like to express our sincere gratitude to the Editor and the Reviewers for their constructive comments and valuable suggestions on our manuscript entitled " An analytical method to optimize residual stress in the ultrasonic rolling of titanium alloys Ti6Al4V" (Manuscript ID: PONE-D-26-00227). These comments are exceptionally helpful for improving the quality of our manuscript. We have carefully considered all comments and made corresponding revisions in the revised manuscript. All changes in the revised manuscript have been highlighted in red color / blue highlight for easy tracking.

Below is our point-by-point response to the reviewers’ comments.

Best regards,

Authors

COMMENTS FOR AUTHORS & ANSWERS

Reviewer #1: The manuscript describes a technically sound piece of scientific research with data that supports the conclusions. Experiments have been conducted rigorously. The conclusions are drawn appropriately based on the data presented.

Additional Clarification on Validation Parameters

The authors sincerely thank the reviewer for this constructive suggestion. In response, the validation section has been substantially expanded and strengthened in the revised manuscript.

First, the authors would like to clarify a textual inconsistency identified in the original manuscript regarding the description of the experimental parameters reported by Bozdana. During preparation of the original manuscript, an earlier draft of the validation section had initially been prepared using parameters associated with a different reference study. Although the validation discussion was subsequently revised to use the experimental residual stress data reported by Bozdana, several parameter values in the descriptive sentence were inadvertently not updated accordingly.

After careful re-examination of the validation procedure, it was confirmed that the actual analytical calculations and residual stress predictions were performed using the correct validation parameters corresponding to the experimental conditions reported by Bozdana, namely a static load of 380 N, a rolling ball diameter of 6 mm, and a vibration amplitude of 10.9 μm. Therefore, this issue was limited to an incorrect textual description in the manuscript and did not affect the analytical calculations, residual stress distributions, response surface analysis, optimization results, or the conclusions of the study. The validation description in the revised manuscript has been corrected accordingly to ensure consistency and clarity.

In addition to the original validation case corresponding to the 380 N ultrasonic deep cold rolling (UDCR) condition reported by Bozdana, an additional validation case corresponding to the 30 N UDCR condition has been incorporated. This additional comparison allows the proposed analytical framework to be evaluated under both low-force and high-force ultrasonic rolling conditions.

Furthermore, quantitative validation metrics including peak stress error, peak depth error, root mean square error (RMSE), and mean absolute error (MAE) were introduced to evaluate the agreement between the analytical predictions and the experimentally observed residual stress profiles. The experimental residual stress data were digitized from the published residual stress profiles using the WebPlotDigitizer software.

The revised results demonstrate that the proposed analytical framework provides reasonable agreement with the experimentally observed residual stress evolution behavior under both validation conditions. In particular, for the 380 N condition, the predicted peak compressive residual stress and corresponding penetration depth show errors of approximately 1.45% and 6.67%, respectively.

The validation section has been corrected in the updated manuscript.

Please review the revised manuscript

Best regards,

Authors

Reviewer #2: Reviewer Comments

The manuscript presents an analytical framework for predicting and optimizing residual stress during ultrasonic rolling of Ti6Al4V. The integration of Hertzian contact theory, elastic–plastic deformation modeling, and response surface methodology is relevant and addresses an important problem in surface engineering and fatigue enhancement of titanium alloys.

The study demonstrates reasonable agreement between predicted and experimental residual stress profiles, with the model capturing both the magnitude and depth of peak compressive stress. This indicates that the proposed analytical formulation has potential practical value. However, several important issues should be addressed before the manuscript can be considered for publication:

Comment 1. Novelty and Contribution

The manuscript does not clearly articulate what is fundamentally new compared to existing analytical and semi-empirical models. The combination of Hertzian contact theory and response surface methodology is well established. The authors should explicitly define the unique contribution of this work and distinguish it from prior studies.

Response

The authors sincerely thank the reviewer for this valuable comment. In response, the novelty and scientific contribution of the present study have been clarified and strengthened in the revised manuscript.

The revised manuscript now explicitly emphasizes that the novelty of the present work does not arise from the isolated use of Hertzian contact mechanics or response surface methodology individually, since these approaches are already well established in the literature. Instead, the main contribution lies in their integration into a unified physics-based analytical framework for predicting and optimizing residual stress evolution during ultrasonic rolling of Ti6Al4V alloy.

Additional discussion has been incorporated to clarify that most previous studies primarily relied on experimental observations or computationally intensive finite-element simulations within relatively limited parameter ranges. In contrast, the proposed framework provides a computationally efficient analytical methodology capable of simultaneously predicting both compressive residual stress magnitude and compressive layer depth under coupled static and ultrasonic loading conditions.

The revised manuscript further clarifies that the proposed approach systematically evaluates the coupled influence of static load, vibration amplitude, ultrasonic frequency, and rolling ball radius on residual stress evolution and hardened layer development through a combined analytical–statistical framework. Consequently, the proposed methodology provides not only physical interpretation of the underlying deformation mechanisms, but also a computationally efficient tool for parametric analysis and process optimization of ultrasonic rolling for Ti6Al4V alloy.

The abstract and introduction section has been updated in the revised manuscript.

Comment 2. Model Validation

Validation is limited to comparison with a single experimental dataset from the literature. This is insufficient for establishing predictive robustness. Additional validation using independent datasets, or at minimum quantitative error metrics (e.g., RMSE, percentage error), should be included.

Response

The authors sincerely thank the reviewer for this constructive suggestion. In response, the validation section has been expanded and revised in the revised manuscript.

First, the authors would like to clarify a textual inconsistency identified in the original manuscript regarding the description of the experimental parameters reported by Bozdana. During preparation of the original manuscript, an earlier draft of the validation section had initially been prepared using parameters associated with a different reference study. Although the validation discussion was subsequently revised to use the experimental residual stress data reported by Bozdana, several parameter values in the descriptive sentence were inadvertently not updated accordingly.

After careful re-examination of the validation procedure, it was confirmed that the actual analytical calculations and residual stress predictions were performed using the correct validation parameters corresponding to the experimental conditions reported by Bozdana, namely a static load of 380 N, a rolling ball diameter of 6 mm, and a vibration amplitude of 10.9 μm. Therefore, this issue was limited to an incorrect textual description in the manuscript and did not affect the analytical calculations, residual stress distributions, response surface analysis, optimization results, or the conclusions of the study. The validation description in the revised manuscript has been corrected accordingly to ensure consistency and clarity.

In addition to the original validation case corresponding to the 380 N ultrasonic deep cold rolling (UDCR) condition reported by Bozdana, an additional validation case corresponding to the 30 N UDCR condition has been incorporated. This additional comparison allows the proposed analytical framework to be evaluated under both low-force and high-force ultrasonic rolling conditions.

Furthermore, quantitative validation metrics including peak stress error, peak depth error, root mean square error (RMSE), and mean absolute error (MAE) were introduced to evaluate the agreement between the analytical predictions and the experimentally observed residual stress profiles. The experimental residual stress data were digitized from the published residual stress profiles using the WebPlotDigitizer software.

The revised results demonstrate that the proposed analytical framework provides reasonable agreement with the experimentally observed residual stress evolution behavior under both validation conditions. In particular, for the 380 N condition, the predicted peak compressive residual stress and corresponding penetration depth show errors of approximately 1.45% and 6.67%, respectively.

The validation section has been expanded and revised in the updated manuscript.

Comment 3. Statistical Modeling Concerns

The response surface models are constructed using data generated from the analytical model itself rather than independent experimental data. This approach can lead to artificially high R² values (reported >0.99) and does not demonstrate true predictive capability. The authors should clarify this limitation and temper claims accordingly.

Response

The authors sincerely thank the reviewer for this important observation. In response, the revised manuscript has been updated to explicitly clarify the limitation associated with the response surface methodology used in the present study.

The revised discussion now clarifies that the response surface models were constructed using data generated from the validated analytical framework rather than from independent experimental measurements. Consequently, the high R² values should primarily be interpreted as indicators of internal consistency and surrogate approximation capability within the investigated parameter domain, rather than generalized predictive capability.

To avoid overstatement, the discussion and conclusions have been revised accordingly. The revised manuscript now emphasizes that the response surface models are intended mainly as computationally efficient tools for parametric analysis and process optimization.

The revised manuscript has been updated accordingly.

Comment 4. Model Assumptions

Several simplifying assumptions are made, including isotropic hardening, neglect of the Bauschinger effect, and simplified superposition of static and dynamic loads. These assumptions may not fully capture the cyclic and high strain-rate nature of ultrasonic rolling. A discussion on the limitations and potential impact of these assumptions is needed.

Response

The authors sincerely thank the reviewer for this insightful comment. In response, an additional discussion regarding the assumptions and limitations of the proposed analytical framework has been incorporated into the revised manuscript.

The revised manuscript now explicitly acknowledges that several simplifying assumptions were adopted to maintain analytical tractability and computational efficiency, including isotropic hardening behavior, neglect of the Bauschinger effect, simplified representation of coupled static and ultrasonic dynamic loading, and omission of strain-rate-dependent constitutive effects and thermal influences.

The revised discussion further clarifies that these simplifications may influence prediction accuracy under highly nonlinear cyclic deformation conditions associated with ultrasonic rolling. Nevertheless, despite these assumptions, the proposed analytical framework still demonstrates reasonable agreement with experimentally observed residual stress profiles and successfully captures the dominant trends governing residual stress evolution.

These limitations were added to the validation section 3.2.1 to clarify the assumptions and constraints associated with the proposed analytical framework.

Comment 5. Physical Interpretation of Results

While parametric trends are presented, the physical explanations remain limited. The authors should provide deeper mechanistic insight into why certain parameters (e.g., static load vs frequency) dominate residual stress formation.

Response

The authors sincerely thank the reviewer for this valuable comment. In response, the discussion of the parametric study has been substantially expanded in the revised manuscript to provide deeper physical interpretation of the observed trends.

Additional mechanistic explanations have been incorporated regarding the effects of static load, ball diameter, vibration amplitude, and ultrasonic frequency on Hertzian contact pressure, cyclic plastic deformation, strain accumulation, and ultrasonic energy transfer during ultrasonic rolling.

The revised discussion further explains static load strongly influences the formation of the subsurface plastic deformation zone because it directly governs the Hertzian contact pressure. In contrast, vibration amplitude mainly enhances cyclic deformation efficiency and ultrasonic energy transfer through repeated dynamic impacts and acoustoplastic effects. The revised discussion also addresses the possibility of plastic deformation saturation at higher static loading conditions, which may increase the relative influence of vibration amplitude during multi-objective optimization.

This additional discussion has been incorporated into the discussion section of the revised manuscript.

6. Clarity and Language

The manuscript requires significant language editing. There are grammatical errors, repetitive phrasing, and sections where clarity is reduced. Improving readability will strengthen the paper considerably.

Response

The authors sincerely thank the reviewer for this helpful comment. In response, the manuscript has undergone extensive language revision to improve clarity, readability, consistency of terminology, and overall academic writing quality.

Grammatical issues, repetitive phrasing, informal wording, and typographical errors throughout the manuscript were carefully corrected. In addition, several sections of the Introduction, Results and Discussion, and Conclusion were revised to improve scientific flow and clarity of presentation.

Particular attention was given to reducing repetitive sentence structures, improving transitions between paragraphs, and refining the physical interpretation of the analytical results. Terminology related to compressive residual stress and hardened layer depth was also standardized throughout the manuscript to improve consistency.

All revised text has been highlighted in red in the revised manuscript.

The authors sincerely appreciate the reviewer’s valuable comments. The revised manuscript has been carefully improved in terms of validation, clarification of novelty, and depth of analysis in accordance with the reviewer’s suggestions.

Best regards,

Authors

Attachment Submitted filename: AnswerSheet_truyen_for_PLOSONE.docx

# Peer review: Decision Letter 1

**DOI:** 10.1371/journal.pone.0351986.r003

**Authors:** Yashwanth Nanda Kumar, Yashwanth Nanda Kumar

4 Jun 2026

An analytical method to optimize residual stress in the ultrasonic rolling of titanium alloys Ti6Al4V

PONE-D-26-00227R1

Dear Dr. le,

We’re pleased to inform you that your manuscript has been judged scientifically suitable for publication and will be formally accepted for publication once it meets all outstanding technical requirements.

Within one week, you’ll receive an e-mail detailing the required amendments. When these have been addressed, you’ll receive a formal acceptance letter and your manuscript will be scheduled for publication.

An invoice will be generated when your article is formally accepted. Please note, if your institution has a publishing partnership with PLOS and your article meets the relevant criteria, all or part of your publication costs will be covered. Please make sure your user information is up-to-date by logging into Editorial Manager at Editorial Manager® and clicking the ‘Update My Information' link at the top of the page. For questions related to billing, please contact billing support.

If your institution or institutions have a press office, please notify them about your upcoming paper to help maximize its impact. If they’ll be preparing press materials, please inform our press team as soon as possible -- no later than 48 hours after receiving the formal acceptance. Your manuscript will remain under strict press embargo until 2 pm Eastern Time on the date of publication. For more information, please contact onepress@plos.org.

Kind regards,

Yashwanth Nanda Kumar, PhD

Academic Editor

PLOS One

Reviewers' comments:

Reviewer's Responses to Questions

**Comments to the Author**

1. If the authors have adequately addressed your comments raised in a previous round of review and you feel that this manuscript is now acceptable for publication, you may indicate that here to bypass the “Comments to the Author” section, enter your conflict of interest statement in the “Confidential to Editor” section, and submit your "Accept" recommendation.

Reviewer #2: All comments have been addressed

**********

2. Is the manuscript technically sound, and do the data support the conclusions?

The manuscript must describe a technically sound piece of scientific research with data that supports the conclusions. Experiments must have been conducted rigorously, with appropriate controls, replication, and sample sizes. The conclusions must be drawn appropriately based on the data presented.

Reviewer #2: Yes

**********

3. Has the statistical analysis been performed appropriately and rigorously?

Reviewer #2: Yes

**********

4. Have the authors made all data underlying the findings in their manuscript fully available?

The PLOS Data policy requires authors to make all data underlying the findings described in their manuscript fully available without restriction, with rare exception (please refer to the Data Availability Statement in the manuscript PDF file). The data should be provided as part of the manuscript or its supporting information, or deposited to a public repository. For example, in addition to summary statistics, the data points behind means, medians and variance measures should be available. If there are restrictions on publicly sharing data—e.g. participant privacy or use of data from a third party—those must be specified.

Reviewer #2: Yes

**********

5. Is the manuscript presented in an intelligible fashion and written in standard English?

PLOS ONE does not copyedit accepted manuscripts, so the language in submitted articles must be clear, correct, and unambiguous. Any typographical or grammatical errors should be corrected at revision, so please note any specific errors here.

Reviewer #2: Yes

**********

6. Review Comments to the Author

Please use the space provided to explain your answers to the questions above. You may also include additional comments for the author, including concerns about dual publication, research ethics, or publication ethics. (Please upload your review as an attachment if it exceeds 20,000 characters)

Reviewer #2: The authors have adequately addressed the major concerns raised during the previous review round. The revised manuscript demonstrates substantial improvement in terms of validation, clarification of novelty, discussion of assumptions and limitations, and overall scientific presentation.

In particular, the addition of quantitative validation metrics (RMSE, MAE, peak stress error, and depth error), incorporation of an additional validation condition, and clarification regarding the limitations of the response surface methodology significantly strengthen the technical rigor and transparency of the study.

The revised manuscript now more clearly defines the contribution of the work as a unified analytical framework integrating Hertzian contact mechanics, elastic–plastic deformation modeling, and response surface methodology for residual stress analysis and process optimization in ultrasonic rolling of Ti6Al4V alloy.

The discussion regarding model assumptions and limitations has also been improved appropriately, and the authors have moderated claims related to predictive capability. Furthermore, the physical interpretation of the parametric trends is more detailed and scientifically justified in the revised version.

While some minor language polishing may still improve readability in certain sections, the manuscript is now technically sound and the conclusions are generally supported by the presented analysis and validation results.

**********

7. PLOS authors have the option to publish the peer review history of their article (what does this mean?). If published, this will include your full peer review and any attached files.

If you choose “no”, your identity will remain anonymous but your review may still be made public.

**Do you want your identity to be public for this peer review?**  For information about this choice, including consent withdrawal, please see our Privacy Policy.

Reviewer #2: **Yes:** Uwais AQ Ahmed

**********

# Editor report: Acceptance letter

**DOI:** 10.1371/journal.pone.0351986.r004

**Authors:** Yashwanth Nanda Kumar, Yashwanth Nanda Kumar

PONE-D-26-00227R1

PLOS One

Dear Dr. le,

I'm pleased to inform you that your manuscript has been deemed suitable for publication in PLOS One. Congratulations! Your manuscript is now being handed over to our production team.

At this stage, our production department will prepare your paper for publication. This includes ensuring the following:

* All references, tables, and figures are properly cited

* All relevant supporting information is included in the manuscript submission,

* There are no issues that prevent the paper from being properly typeset

You will receive further instructions from the production team, including instructions on how to review your proof when it is ready. Please keep in mind that we are working through a large volume of accepted articles, so please give us a few days to review your paper and let you know the next and final steps.

Lastly, if your institution or institutions have a press office, please let them know about your upcoming paper now to help maximize its impact. If they'll be preparing press materials, please inform our press team within the next 48 hours. Your manuscript will remain under strict press embargo until 2 pm Eastern Time on the date of publication. For more information, please contact onepress@plos.org.

You will receive an invoice from PLOS for your publication fee after your manuscript has reached the completed accept phase. If you receive an email requesting payment before acceptance or for any other service, this may be a phishing scheme. Learn how to identify phishing emails and protect your accounts at https://explore.plos.org/phishing.

If we can help with anything else, please email us at customercare@plos.org.

Thank you for submitting your work to PLOS ONE and supporting open access.

Kind regards,

PLOS ONE Editorial Office Staff

on behalf of

Dr. Yashwanth Nanda Kumar

Academic Editor

PLOS One
